# Emerin mislocalization during chromatin bridge resolution can drive prostate cancer cell invasiveness in a collagen-rich microenvironment

**DOI:** 10.1038/s12276-024-01308-w

**Published:** 2024-09-02

**Authors:** Marta Popęda, Kamil Kowalski, Tomasz Wenta, Galina V. Beznoussenko, Michał Rychłowski, Alexander Mironov, Zeno Lavagnino, Sara Barozzi, Julia Richert, Rebecca Bertolio, Kamil Myszczyński, Jolanta Szade, Michał Bieńkowski, Kevin Miszewski, Marcin Matuszewski, Anna J. Żaczek, Luca Braga, Giannino Del Sal, Natalia Bednarz-Knoll, Paolo Maiuri, Paulina Nastały

**Affiliations:** 1 Division of Translational Oncology, Intercollegiate Faculty of Biotechnology, University of Gdańsk and Medical University of Gdańsk, Gdańsk, Poland; 2 Department of Pathomorphology, Medical University of Gdańsk, Gdańsk, Poland; 3 Department of General and Medical Biochemistry, Faculty of Biology, University of Gdansk, Gdansk, Poland; 4 IFOM ETS—The AIRC Institute of Molecular Oncology, Milan, Italy; 5 Laboratory of Virus Molecular Biology, Intercollegiate Faculty of Biotechnology, University of Gdansk and Medical University of Gdansk, Gdansk, Poland; 6 International Centre for Genetic Engineering and Biotechnology (ICGEB), Area Science Park-Padriciano, Trieste, Italy; 7 Centre of Biostatistics and Bioinformatics Analysis, Medical University of Gdansk, Gdansk, Poland; 8 Department of Urology, Medical University of Gdańsk, Gdańsk, Poland; 9 Department of Life Sciences, University of Trieste, Trieste, Italy; 10 Department of Molecular Medicine and Medical Biotechnology, University of Naples Federico II, Naples, Italy

**Keywords:** Nuclear envelope, Prostate cancer, Metastasis

## Abstract

Micronuclei (MN) can form through many mechanisms, including the breakage of aberrant cytokinetic chromatin bridges. The frequent observation of MN in tumors suggests that they might not merely be passive elements but could instead play active roles in tumor progression. Here, we propose a mechanism through which the presence of micronuclei could induce specific phenotypic and functional changes in cells and increase the invasive potential of cancer cells. Through the integration of diverse in vitro imaging and molecular techniques supported by clinical samples from patients with prostate cancer (PCa) defined as high-risk by the D’Amico classification, we demonstrate that the resolution of chromosome bridges can result in the accumulation of Emerin and the formation of Emerin-rich MN. These structures are negative for Lamin A/C and positive for the Lamin-B receptor and Sec61β. MN can act as a protein sinks and result in the pauperization of Emerin from the nuclear envelope. The Emerin mislocalization phenotype is associated with a molecular signature that is correlated with a poor prognosis in PCa patients and is enriched in metastatic samples. Emerin mislocalization corresponds with increases in the migratory and invasive potential of tumor cells, especially in a collagen-rich microenvironment. Our study demonstrates that the mislocalization of Emerin to MN results in increased cell invasiveness, thereby worsening patient prognosis.

## Micronuclei induce cell invasiveness in prostate cancer progression

Micronuclei, small nuclei formed during incorrect cell division, can signal genomic instability, often found in cancer. This research investigates how micronuclei may contribute to cancer progression, in the context of prostate cancer, by focusing on the nuclear envelope protein, Emerin. The team conducted experiments using different cell lines and patient samples to understand how Emerin’s misplacement from nuclear envelope to micronuclei can impact cancer cell behavior. The study involved analyzing cells for Emerin distribution, observing the effects of Emerin misplacement on cell movement and invasiveness, and examining prostate cancer tissue to see how these findings relate to the disease in patients. It was observed that the presence of Emerin-rich micronuclei correlates to more aggressive cancer behavior and could predict worse outcomes. Targeting the pathways leading to Emerin misplacement could offer novel strategies for treating aggressive prostate cancer.

This summary was initially drafted using artificial intelligence, then revised and fact-checked by the author.

## Introduction

Micronuclei (MN), which are markers of chromosomal instability, can be defined as small, chromatin-containing nuclear structures that are physically separated from the main nucleus of a cell^1^. Their structure and nuclear envelope (NE) composition differ significantly from those of the primary nucleus^1–3^. MN can be formed from lagging chromosomes or chromosome fragments resulting from mitotic errors or DNA damage coupled with chromatin bridge breakage^4,5^. They have long been used as biomarkers of genotoxicity, tumor risk, and tumor grade^5–7^. However, the frequent observation of micronuclei in tumors raises the possibility that they might not be merely passive warnings but instead could play active roles in tumor progression, e.g., by inducing chromothripsis^2^ or by activating innate immune signaling pathways^8,9^. It has never been shown whether the presence of micronuclei can lead to protein mislocalization or, consequently, induce phenotypic and functional changes in cells, leading to increased invasive potential. Recent findings suggest that the composition of proteins at the NE is uneven throughout the membrane and that zones with the accumulation of some proteins, e.g., the inner and outer nuclear membrane protein Emerin, can be formed in relation to cell polarization^10,11^.

Emerin is a relatively small (~25 kDa) protein that contains a hydrophobic (transmembrane) domain near the C-terminus and a very small luminal domain^12,13^. Consistent with this domain organization, newly synthesized Emerin polypeptides in the cytoplasm are inserted into the endoplasmic reticulum (ER) membrane posttranslationally. After insertion, these polypeptides diffuse throughout the contiguous membranes of the ER/NE, including the nuclear pore complex membranes, where the outer and inner NE membranes are connected^14^. Emerin mislocalization was previously reported in prostate cancer (PCa)^15^, which is the second most common cancer in men and remains a leading cause of death worldwide^16^. Chromosomal instability in PCa has been linked to a higher Gleason score^17^ and increased metastatic potential, leading to greater lethality^18–20^. In the present study, we propose that the accumulation of Emerin in the micronuclei of PCa cells can lead to its consequent pauperization at the NE, increasing the migratory and invasive potential of PCa cells. We also report that such a phenotype is more frequently found in metastatic PCa samples and can be associated with a collagen-rich microenvironment in this tumor type.

## Material and methods

### Cell lines

The human immortalized prostate epithelial cell line RWPE-1 (CRL-3607) was purchased from ATCC (American Type Culture Collection) and cultured in keratinocyte serum-free medium (Thermo Fisher #17005042) supplemented with 1% penicillin‒streptomycin (Thermo Fisher Scientific #15140122). The human prostate cancer cell lines DU145 (HTB-81), LNCaP (CRL-1740), and PC-3 (#CRL-1435) were purchased from ATCC and cultured in RPMI 1640 medium (Thermo Fisher Scientific #11875093) supplemented with 10% FBS (Thermo Fisher Scientific #10270-106) and 1% penicillin‒streptomycin (Thermo Fisher Scientific #15140122). The cell lines were periodically tested for mycoplasma contamination. All cell lines were used for no more than 5-10 passages after thawing.

### Patient samples and ethical approval

The study was approved by the local ethics committee (i.e., Independent Bioethics Committee for Scientific Research at the Medical University of Gdańsk, no. NKBBN/286/2018). Written informed consent was obtained from all subjects involved in the study. A total of 351 primary tumor samples from 117 patients with PCa defined as intermediate- to high-risk by the D’Amico classification who underwent radical prostatectomy in the Department of Urology at the Medical University of Gdańsk (Poland) between 2018 and 2022 were analyzed. Tumor specimens were prepared as tumor microarrays (TMAs) that consisted of 3 tumor fragments per patient and included fragments of tumors with the highest and most common Gleason scores^21^. The percentage of tumor cells in all the tissue cores was determined by an experienced pathologist. The clinical data of the patients are presented in Supplementary Table 1.

### Metastatic samples

The cohort comprised 14 patients with metastatic prostate cancer who underwent a biopsy of a metastatic site or metastasectomy at the University Clinical Center in Gdańsk between 2013 and 2023. All specimens were subjected to pathologic review, and the most representative areas were selected for TMA construction, with 2 cores (1.5 mm in diameter) sampled from each metastatic focus. The percentage of tumor cells per core was determined by a pathologist. Details on the locations of the metastatic foci and the type of tissue sampling performed are provided in Supplementary Table 5.

### Immunofluorescence staining of cells

The selected antigens were immunolocalized in cultured cells plated on collagen-coated coverslips. The cells were fixed with 4% PFA for 10 min, washed with 1× PBS, and permeabilized with 0.1% Triton X-100/1× PBS (Sigma‒Aldrich). Non-specific binding of antibodies was blocked with 3% bovine serum albumin (BSA) in 1× PBS at room temperature for 1 h. Then, the samples were incubated overnight with primary antibodies (Supplementary Table 7) diluted in PBS containing 3% BSA, washed with 1× PBS, and incubated at room temperature with secondary antibodies diluted 1:500 in 1× PBS containing 1.5% BSA. After washing with 1x PBS, cell nuclei were visualized by staining with DAPI (Thermo Fisher Scientific #D1306). The specimens were mounted with ProLong™ Gold Antifade Mounting Medium (Thermo Fisher Scientific #P36930).

### Immunofluorescence staining of tissues

First, 4- to 6-µm-thick TMA sections were deparaffinized, and antigen retrieval was performed using EnVision FLEX Target Retrieval Solution Low pH (Dako Agilent) in a PT Link device (PT200, Dako Agilent) for 20 min at 97 °C. The TMAs were then incubated overnight at 4 °C with a mouse monoclonal anti-Emerin in vitro diagnostic antibody (clone 4G5, Novocastra) diluted 1:100 and/or an anti-Lamin B1 antibody diluted 1:100 (Abcam, ab16048) prior to incubation with a secondary antibody for 60 min. Nuclei were stained with DAPI, and the sections were mounted with Vectashield Antifade Mounting Medium (Vector Laboratories).

### Picrosirius Red staining and collagen fiber quantification

Collagen fibers were visualized with a Picro-Sirius Red Stain Kit (Connective Tissue Stain) (Abcam, ab150681) according to the manufacturer’s instructions. The area of collagen fibers was manually marked and presented as the percentage of the total collagen fiber area as determined using ImageJ software^22^.

### Quantification of Emerin-rich MN in PCa tissue, metastatic PCa tissue, and cell samples

To determine the number of Emerin-rich MN/nuclei, a custom integrated image analysis workflow combining QuPath^23^ and ImageJ^24,25^ was developed. The positions of the prostate cancer cells were selected. The presence of tumor cells was verified by an experienced pathologist. The immunohistochemical stain AMACR^26^ was used as a confirmatory stain for prostatic adenocarcinoma in combination with H&E staining for morphological evaluation. The number of nuclei in the selected region was determined using the cell detection tool in QuPath software (ver. 0.3.0). The selected region was further imported into ImageJ (ver. 2.3.0), where data for individual fluorescence channels were loaded into the workflow and annotated, and the nuclear and Emerin images were processed by parallel segmentation. The Emerin images were segmented by conventional thresholding and converted to a binary mask using ImageJ. Emerin-rich MN were subsequently identified (on the basis of their size (0.2–12 µm^2^) and Emerin staining intensity). The segmentation models were improved through an iterative training process until ≥95 concordance was achieved with manual counting. For each field of view, Emerin-rich MN was identified and counted using the ImageJ Analyze Particles function (https://imagej.net/imaging/particle-analysis). The identification and quantitation of Emerin-rich micronuclei in cells were performed with a similar workflow but without region selection in QuPath. Each field of view was then verified by an independent observer. Emerin expression was stratified with the following cutoff criteria: <25% percentile: <0.07538 for Emerin-rich MN/nuclei; median: 0.1280 for Emerin-rich MN/nuclei; and >75% percentile: 0.1861). Emerin-negative (denoting pauperized Emerin) PCa samples were defined as those in which >85% of the tumor cells exhibited negative staining or low staining intensity ( + 1) for Emerin.

### Superresolution stimulated emission depletion (STED) microscopy

For STED imaging^27^, cells were fixed with 4% PFA, blocked with 10% normal goat serum (Thermo Fisher Scientific), and incubated for 45 min with anti-Emerin (clone 4G5, Novocastra) and anti-Lamin B1 (Abcam, ab16048) antibodies. Then, the cells were incubated with the following secondary antibodies: anti-mouse IgG Atto-594 (Sigma‒Aldrich, 76085) and anti-rabbit IgG-Atto-647N (Sigma‒Aldrich, 40839). Each specimen was then mounted in Mowiol (Sigma‒Aldrich). The cells were imaged with an HC PL APO 100×1.4 NA STED oil immersion objective (Leica Microsystems) mounted on a Leica TCS SP8 STED microscope (Leica Microsystems) equipped with a tunable pulsed white light laser source for confocal imaging and with depletion lasers (592 nm—continuous wave and 775 nm—pulsed).

### Correlative light electron microscopy (CLEM)

PC-3 cells were seeded into collagen-coated 35 mm glass bottom dishes with grids (MatTek Life Sciences) and incubated for 48 h to allow attachment. The cells were fixed for 5 min by incubation with 4% PFA/0.05% glutaraldehyde/0.15 M HEPES (pH 7.3) buffer followed by incubation with the same buffer (3×) for 10 min. The specimens were blocked for 20 min in 1% BSA/PBS and then permeabilized for 15 min in 0.1% saponin/1× PBS. The cells were incubated with a mouse anti-Emerin (clone 4G5, Novocastra) primary antibody for 45 min and then with an anti-mouse secondary antibody conjugated to Alexa Fluor 488 (Thermo Fisher Scientific) for 45 min. Bright-field and fluorescence images of the stained cells were immediately acquired with a Leica CLSM TCS SP2 inverted microscope equipped with 20x (air, 0.55 NA) and 40x (air, 0.95 NA) objectives. Two-step CLEM based on the analysis of tomographic reconstructions acquired at low magnification with consecutive reacquisition of the EM tomography box at high magnification and re-examination of the box was performed exactly as previously described^28^. Briefly, an ultramicrotome (Leica EM UC7; Leica Microsystems) was used to slice 200-nm serial semithick sections. The sections were mounted on 1% Formvar-coated films adhered to slot grids. Both sides of the grids were labeled with protein A-coated 10 nm gold particles (PAG10, CMC). Tilt series were acquired for the samples from ±65° with 1° increments at 200 kV via Tecnai 20 electron microscopes (FEI, Thermo Fisher Scientific). Tilt series were acquired at a magnification of 19,000x using the software supplied with the instrument. The nominal resolution in our tomograms was 4 nm and was based on the section thickness, number of tilts, tilt increments, and tilt angle range. The IMOD package and its newest viewer, 3DMOD 4.0.11, were used to construct individual tomograms, and for the assignment of the outer leaflets of organelle membrane contours, CLEM was performed exactly as previously described^29^.

### Generation of stable EGFP-EMD PC-3 cells

PC-3 cells were transfected with Emerin-pEGFP-C1 (Addgene plasmid #61993). Forty-eight hours post-transfection, the cells were cultured in a medium supplemented with G-418 (400 μg/mL, Life Technologies, Cat. 11811-031) for 1 week. Afterward, single cells were plated onto 96-well plates. Colonies obtained from single cells expressing green fluorescence were selected and further propagated.

### Microscopy

Widefield fluorescence microscopy was performed with a Zeiss Axio Observer 7 equipped with an Axiocam 506 monochromatic camera with a 20x objective (Plan-Neofluar 20x/0.50). Z-stack images were acquired with a confocal laser scanning microscope (TCS SP8; Leica Microsystems) at a distance of 0.3-0.5 μm from the focal plane using the z-stack function. The following oil immersion objectives were used: 20x oil immersion HC PL APO 0.75 NA and 63x oil immersion HC PL APO 1.4 NA objectives (Leica Microsystems). The microscope was equipped with Leica Application Suite X software (ver. 3.5.7.23225).

### Video microscopy

Video microscopy was performed with a Leica TCS SP8x microscope using incubation chambers and gas controllers from Life Imaging Services GmbH (Switzerland). PC-3 cells stably transfected with the Emerin-pEGFP-C1 plasmid (Addgene, #61993) were allowed to attach to collagen-coated 35 mm glass bottom dishes (MatTek Life Sciences) for 48 h. The cells were imaged for 16–48 h at 5 min intervals. The following oil immersion objectives were used: 20x oil immersion HC PL APO 0.75 NA and 63x oil immersion HC PL APO 1.4 NA objectives (Leica Microsystems). The microscope was equipped with Leica Application Suite X software (ver. 3.5.7.23225).

### siRNA transfection and drug treatment

The knockdown of selected target genes was performed using siRNA (Supplementary Table 8) with RNAiMAX (Thermo Fisher Scientific #13778150) reagent following the manufacturer´s instructions. The cells were cultured for 72 h after transfection. The cells were treated with 5 μM ICRF-193 (ChemCruz # CAS 21416-68-2) for 24 h before analysis. A CENP-E inhibitor (MCE, #GSK-923295) was added at a concentration of 100 nM for 8 h, after which a washout step was performed. Palbociclib (Sellecktchem,# PD 0332991) was used at a concentration of 1 μM for 72 h. Brefeldin A (Thermo Fisher Scientific #B7450) was used at concentrations of 0.3 and 2.5 μM for 16 h.

### Western blot analysis

Proteins were extracted by cell lysis using RIPA buffer (Thermo Fisher Scientific #89901) containing a protease inhibitor (Thermo Fisher Scientific #78438). Proteins in the cleared lysates were separated by SDS‒PAGE and transferred to nitrocellulose membranes. The membranes were incubated in 5% skim milk in TBS-T as a blocking reagent and were then incubated with primary antibodies (Supplementary Table 7) and the appropriate fluorochrome-conjugated secondary antibodies. Protein bands were visualized on an Odyssey CLx Imager.

### Gene knockout

Lentivirus-CRISPR/Cas9-mediated gene knockout was performed as described previously^30^. Two independent, specific sequences targeting constitutive early exons of Emerin (EMD) were designed (EMD1-TTGTACCGGCGCAGCAAGG, targeting exon 1; EMD2- TCTTCGAGTACGAGACCCAG, targeting exon 2). To prevent off-targeting, the specificity of the gRNA sequences was validated using the FASTA similarity search tool (EMBL-EBI) (criterion: more than three mismatches with any other site in the human genome (GRCh38.p13). The EMD1 and EMD2 gRNA sequences were inserted into the BsmBI restriction sites in the plentiCRISPRv2_puro vector (Addgene, 52961), and the constructed plasmids were used for lentivirus preparation. Lentiviral particles were generated by cotransfecting 5 μg of the specific pLentiCRISPRv2 plasmid, 3.75 μg of psPax2, and 1.25 μg of pVSV-G (a second-generation lentivirus packaging system) into the human embryonic kidney packaging cell line GP2-293 (Clontech, Inc.) using a CalPhos™ Mammalian Transfection Kit (Clontech, Inc.) according to the manufacturer’s protocol, as previously described^31^. Successfully transduced cells were selected by incubation with 1 μg/mL puromycin for at least 10 days, and protein expression was then analyzed by western blotting.

### Inverse fluorescence recovery after photobleaching (iFRAP)

PC-3 prostate cancer cells expressing EGFP-EMD were incubated for 48 h to allow attachment to collagen-coated 35 mm glass bottom dishes (MatTek Life Sciences). The cells were imaged using an Olympus Spinning Disk CSU system based on an Olympus IX83 inverted microscope equipped with an Andor iXon Ultra camera. Images were acquired with a U PLAN S APO 60x/1.35 NA oil immersion objective using CellSens software (Olympus). NEs and Emerin-rich structures were photobleached using a 405 nm laser at maximum power. The cells were imaged before and 7 min after photobleaching within a 5 s timeframe. During the experiment, the cells were maintained at 37 °C in a humidified atmosphere with 5% CO_2_ using an incubator system (Okolab).

### DAPI content quantification

For analysis of the differential signal intensity in nuclei and micronuclei, images were acquired, and nuclei and micronuclei were identified based on the signals in the DAPI and Emerin channels, respectively. The DAPI signal was segmented using an Otsu filter (value = 25). The mean signal intensity of the region of interest, including the nucleus and micronucleus, was compared, and the ratio of the two signal intensities was calculated.

### NE pauperization quantification

For analysis of NE pauperization, images were acquired, cells with and without Emerin-rich MN were identified based on the signal in the Emerin channel, and the signal was segmented using an Intermodes filter (value = 25). The mean signal intensity of the region containing the whole NE was calculated and divided by the median intensity of the cytoplasmic membrane signal in cells without MN, in cells with Emerin-rich MN, and in cells after BFA treatment.

### Nuclear area measurement

Nuclei were identified as DAPI-positive areas, and the signal was segmented using an Otsu filter. The area and shape parameters were calculated for the structures identified and counted using the ImageJ Analyze Particles function (https://imagej.net/imaging/particle-analysis).

### F-actin coherency

F-actin coherency was calculated from the projected confocal images using the Orientation J plugin (http://bigwww.epfl.ch/demo/orientationj/) for ImageJ^32^, with the F-actin fiber coherency parameter calculated for selected cells.

### Focal adhesion size analysis

To visualize focal adhesions, cells were stained with an anti-paxillin antibody. Using a custom ImageJ macro^11^^,^ the best z-plane was selected, the signal was segmented using Intermodes (value 25), and the size, number, and shape descriptors of the particles were determined. For each cell, the average area of the focal adhesions was calculated.

### Quantification of the MTOC-nucleus distance

Immunofluorescence staining using an anti-pericentrin antibody was performed, and nuclei were visualized using DAPI. After Z-stack projection, the nuclei were registered to the reference nucleus using the turboreg ImageJ plugin. For each channel, all the registered images were combined in a single stack. Then, the stack was segmented using the threshold value for each image in the stack, with Yen (value = 25) used for the pericentrin signal and Otsu (value = 100) used for the nuclear signal. Finally, the custom ImageJ macro^11^ was used to measure the distance between the centrosome and the closest border of the nucleus for each cell. A value < 0 indicated that the centrosome was positioned above the nucleus.

### NanoString nCounter gene expression assay

Analysis of differentially expressed genes based on the Emerin status was performed as previously described^21^. Briefly, total RNA was extracted from 69 samples prepared as formalin-fixed paraffin-embedded (FFPE) primary prostate tumor tissue cores (three 20 µm-thick, unstained FFPE sections per patient) using an RNeasy Mini Kit (QIAGEN, Hilden, Germany) according to the manufacturer’s protocol. RNA integrity was assessed using an Agilent 2100 Bioanalyzer (Agilent Technologies, Santa Clara, CA, USA) with an Agilent RNA 6000 Pico Kit (Agilent Technologies). Extracted RNA (4 µL) was preamplified using the nCounter Low RNA Input Kit (NanoString Technologies, Seattle, WA, USA) with a dedicated primer pool covering the sequences of 740 genes included in the nCounter Cancer Progression Profiling Panel (NanoString Technologies). Preamplified samples were analyzed using the NanoString nCounter Analysis System (NanoString Technologies) according to the manufacturer’s procedures for hybridization, detection, and scanning.

Background correction and data normalization were performed using nSolver 4.0 software (NanoString Technologies). The background level was estimated by thresholding over the mean plus 2 standard deviations of negative control counts, and the data were normalized according to the global mean of the counts of the positive control probes included in the assay and the 4 most stably expressed housekeeping genes, namely, *CNOT4*, *HDAC3*, *DDX50*, and *CC2D1B*. Sixty-six primary prostate tumor samples were available for Emerin imaging analysis (uninformative samples or samples damaged during processing were excluded from the analyses), and their transcriptomic profiles were further correlated with the Emerin status. The cutoff criteria used to evaluate the Emerin status were as follows: <25% percentile: <0.07538 for Emerin-rich MN/nuclei; median: 0.1280 for Emerin-rich MN/nuclei; and >75% percentile: 0.1861. Emerin-negative (denoting pauperized Emerin) PCa samples were defined as those in which >85% of the tumor cells exhibited negative staining or a low staining intensity ( + 1) for Emerin.

### Cell culture on hydrogels

Cells were plated on soft (0.5 kPa) or stiff (50 kPa) collagen I-coated hydrogel plates with glass bottoms (Softwell, Atlantis Bioscience) and on glass coverslips coated with collagen I. After 48 h of incubation, the cells were fixed and stained.

### Spheroid culture

Single PC-3 cells were resuspended in growth factor-reduced solubilized basement membrane (Matrigel®, Corning) mixed with Collagen I (Corning® Collagen I, High Concentration, Rat Tail) to a concentration of 5 mg/mL and plated dropwise in 8-well glass bottom ibidi chambers (ibidi) at 8000 cells/100 μL. The cells were grown for 7 days in RPMI 1640 medium (Thermo Fisher Scientific #11875093) supplemented with 10% FBS (Thermo Fisher Scientific #10270-106), and the medium was changed every 2 days. The spheroids were fixed with 4% PFA, incubated overnight with primary antibodies diluted in 0.5% Triton X-100/5% FBS for staining, and incubated with secondary antibodies in the same buffer. The single spheroids were then imaged via confocal microscopy. Invading cells were defined as cells in which the nucleus was positioned outside of the main cell body. The properties of the spheroids, including the area, circularity, and solidity, were quantified using ImageJ.

### RNA sequencing

Total RNA from spheroids was extracted using a PureLink™ RNA Mini Kit (Thermo Fisher Scientific) following phenol/chloroform purification according to the manufacturer’s instructions following phenol/chloroform purification (Thermo Fisher Scientific), and RNA purity and concentration testing were performed using a NanoDrop 2000 spectrophotometer (Life Technologies). An Agilent RNA ScreenTape System (Agilent) was used for RNA integrity analysis. RNA sequencing libraries were generated according to the manufacturer’s instructions for TruSeq Total RNA with RiboZero Human/Mouse/Rat Gold (Illumina). Sequencing was then performed on the NovaSeq 6000 (Illumina) platform.

### Time-lapse microscopy and migration analysis

Prostate cancer PC-3 cells transfected with the Emerin-pEGFP-C1 plasmid (Addgene, #61993) were imaged using an Operetta CLS microscope in confocal mode with a 40× water immersion lens (NA 1.1). Images were acquired every 7 min for 16 h. The cells were then manually tracked using the ImageJ TrackMate plugin^33^.

### Transwell assay

The membranes of Transwell inserts (pore size of 8.0 μm, Corning) were coated with collagen I (Corning® Collagen I, High Concentration, Rat Tail) for migration assays. Briefly, 1 × 10^5^ cells were resuspended in 500 μL of serum-free medium and seeded in the top compartment of a Transwell chamber. Medium supplemented with 10% FBS (five hundred microliters per well) was added to a 12-well plate. The Transwell inserts were placed in the plate, which was incubated for 24 h. The number of Emerin-rich MN/cells that migrated through the pores was quantified by imaging using at least 4 randomly chosen fields/experiments.

### Collagen invasion assay

PC-3 cells were seeded at the optimal density (30,000 cells/well) and allowed to adhere before removal of the medium. Then, the cell layer was overlaid with 40 μL of Collagen I (Corning® Collagen I, High Concentration, Rat Tail) at a concentration of 5 mg/mL. Fresh RPMI 1640 medium (Thermo Fisher Scientific #11875093) supplemented with 10% FBS (Thermo Fisher Scientific #10270-106) was added, and cell invasion was assayed after incubation for 48 h by fixing cells with 4% PFA and then performing IF staining. The number of Emerin-rich MN/cells that invaded the collagen layer was quantified by imaging using at least 5 randomly chosen fields/experiments and compared to the number of cells that did not invade the gel.

### TCGA and cBioPortal data

The Cancer Genome Atlas (TCGA) prostate adenocarcinoma (PRAD) dataset containing the clinical characteristics and RNA-seq data (RNASeqV2, RSEM normalized) covering the normalized counts of sequences aligning to 20,531 genes for 497 PRAD patients were obtained from the cBioPortal for Cancer Genomics resource^34^ (Firehose Legacy dataset, data access: 26th July 2022). The methods used for biospecimen procurement, RNA isolation, and RNA sequencing were previously described by the TCGA Research Network^35^. *EMD* mutation data were obtained from the cBioPortal for Cancer Genomics (https://cbioportal.org/), where 1,607 prostate adenocarcinoma samples profiled for mutations in the *EMD* gene were available (data access: 6th of September 2023).

### Bioinformatic analyses

#### RNA expression in PC-3 cells

See Supplementary Information: Material and Methods.

#### PCa tumors

In the nCounter gene expression dataset, differentially expressed genes (DEGs) were identified based on the criterion *p* < 0.05 (Mann‒Whitney–Wilcoxon test), and median-based log2FC values for the pauperized vs. normal Emerin comparison were reported. DEGs were associated with GO BP (Gene Ontology biological process) terms using the Functional Annotation Tool of DAVID Bioinformatics Resources 6.8^36,37^, and the interaction network of their protein products was visualized using STRING v11^38^. In the TCGA PRAD cohort, the pauperized Emerin transcriptomic score was calculated as the mean of the log2 RSEM-normalized counts of 9 genes (*CXCR4*, *APOE*, *SPARC*, *VIM*, *GSN*, *ANXA2P2*, *SFRP1*, *COL18A1*, and *FN1*) in each sample. The Emerin pauperization status was classified based on the upper quartile of the score.

### Statistical analysis and reproducibility

All the data are presented as scatter plots or box plots and are expressed as the means ± SDs unless otherwise indicated. The number of experiments and the number of samples analyzed are specified for each experiment, and additional methods used to calculate these parameters are described in the figure legends. Statistical significance was calculated for all instances in which we compared two distinct distributions using the parametric two-tailed unpaired Student’s *t*-test, with an unpaired *t*-test, paired *t*-test or one-way ANOVA with Dunnett’s test or the appropriate correction or the nonparametric two-tailed Mann–Whitney *U-*test used as indicated. The Kruskal–Wallis test with Dunn’s test was used for one-way analysis of variance for comparisons among more than two groups. Paired analysis was performed using the Wilcoxon matched-pairs signed-rank test. Contingency analysis was performed using Fisher’s exact test. Correlations between numeric data were assessed using the Kendall rank correlation coefficient. Associations between the frequency of Emerin-rich MN and time-to-biochemical recurrence were evaluated using the log–rank test, and the data are presented as Kaplan–Meier curves. *p < 0.05, **p < 0.01, ***p < 0.001, ****p < 0.0001. Statistical analysis of quantitative data was carried out in GraphPad Prism (v. 9.0.0) or in R (version 4.2.2) using R statistical packages. Graphical representations were prepared with BioRender.

## Results

### Emerin-rich MN are prevalent in prostate cancer and are correlated with poor prognosis

**Fig. 1:**
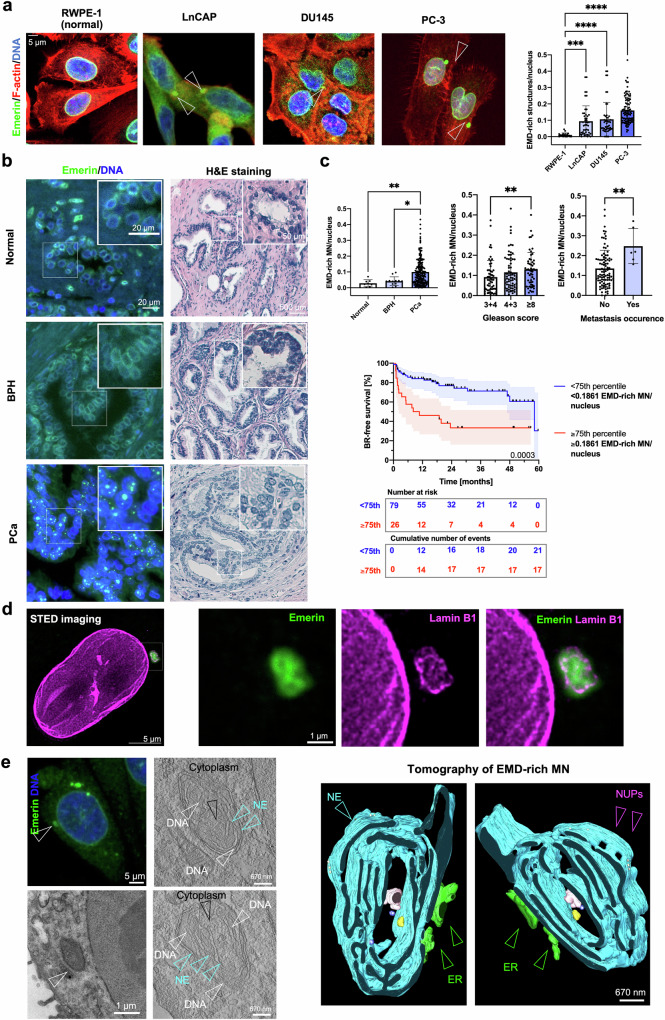
Emerin-rich (EMD-rich) MNs show characteristic ultrastructures and are prevalent in PCa, where they correlate with more aggressive disease. **a** Distribution of Emerin-rich MN in a normal prostate epithelial cell line (RWPE-1, *n* = 20 fields of view, containing >250 cells) and prostate cancer cell lines—LNCaP (*n* = 46 fields of view, containing >250 cells), DU145 (*n* = 48 fields of view, containing >250 cells), and PC-3 (*n* = 98 fields of view, containing >250 cells). **b** Distribution of Emerin-rich MN in normal prostate tissue (*n* = 4 individuals), benign prostatic hyperplasia tissue (BPH patients, *n* = 8), and prostate cancer tissue (PCa, *n* = 221 tissue cores) with corresponding H&E staining. **c** Clinical associations between Emerin-rich MN detected within the primary tumor and the Gleason score (*n* = 180 tissue cores), the development of metastasis after prostatectomy (*n* = 107), and the time to biochemical recurrence (*n* = 105 patients). **d** Superresolution stimulated emission depletion (STED) microscopy images of Emerin-rich MN stained for Emerin and Lamin B1. Emerin is shown in green, and Lamin B1 is shown in magenta. **e** Correlative light electron microscopy (CLEM) images of Emerin-rich MN. Upper left panel: Fluorescence confocal microphotography and associated electron microscopy images. Right panel: tomograms of Emerin-rich MN. NUPs: nuclear pores. Kaplan–Meier curves: log-rank test; the error bars indicate the 95% CIs. Scatter plot with bar (mean with SD): Mann‒Whitney *U*-test or, for comparisons among more than two groups, Kruskal–Wallis test. The error bars indicate the SDs. *p < 0.05; **p < 0.01; ***p < 0.001; ****p < 0.0001; ns, not significant.

Emerin, an inner and outer nuclear membrane protein^11^, has been reported as one of the “core” NE proteins found in micronuclei and on lagging chromosomes^2,3^. Intriguingly, a variety of PCa cell lines (LNCaP, DU145 and PC-3) often harbor micronuclei that exhibit extremely bright staining for Emerin and are very rarely detected in the normal prostate cell line RWPE-1 (Fig. 1a). In such structures, the Emerin staining intensity was nearly twice that at the NE (Supplementary Fig. 1a), where the protein is expected to localize. We further referred to these structures as Emerin-rich MN. The presence of Emerin-rich MN was not associated with differences in the total protein level of Emerin in these cell lines (Supplementary Fig. 1b). To test whether Emerin-rich MN are genuine cancer-associated structures or are an artifact in some cancer cell lines, we performed immunofluorescence staining of samples of PCa defined as high-risk by the D’Amico classification (Supplementary Table 1). In confirmation of our results in cancer cell lines, we detected very bright (with a fluorescence intensity exceeding that of Emerin at the NE) Emerin -rich structures (0-0.4315; 25th to 75th percentile: 0.07538–0.1861, median: 0.1280; Supplementary Fig. 1c) with a much greater frequency in PCa tissue than in benign prostatic hyperplasia (BPH) tissue and normal prostate tissue (Fig. 1b). Most importantly, greater numbers of Emerin-rich structures were correlated with higher Gleason scores (a prostate cancer grading system), the presence of metastasis after radical prostatectomy and poorer patient prognosis (expressed as time to biochemical recurrence, i.e., time to an increase in the prostate-specific antigen (PSA) level after radical prostatectomy) after radical prostatectomy ( ≥ 75th percentile vs. <75th percentile: HR = 3.13, 95% CI: 1.64–5.98, Cox *p*-value = 0.0006; Fig. 1c). No correlations with T status, tumor size or preoperative serum PSA level were found (Supplementary Fig. 1d). Multivariate analysis revealed that along with the Gleason score, Emerin-rich structures were prognostically significant in predicting biochemical recurrence (Supplementary Table 2).

To investigate whether this pattern of staining could be due to *EMD* mutations or changes in gene expression, we analyzed data from public repositories (The Cancer Genome Atlas and cBioPortal for Cancer Genomics). We found that the *EMD* gene is very rarely mutated ( < 0.4% of 1607 cases of prostate adenocarcinoma) and that its expression increases slightly with increasing Gleason score (Supplementary Fig. 1e). Its expression is also slightly greater in castration-resistant PCa tissues than in primary tumors^39^ (Supplementary Fig. 1e). Thus, since Emerin deregulation is associated with increased metastatic potential^15,40,41^ but no frequent mutations or significant changes in gene expression were found in cancer patients, alternative the presence of Emerin at the NE might be modulated through other mechanisms, e.g., through its aberrant localization^15^.

### Emerin-rich MN has distorted NE membranes

We then investigated the fine structure of Emerin-rich MN via superresolution microscopy (STED) and correlative light electron microscopy (CLEM). Using STED, we observed that Emerin-rich MN contained disrupted sheets of lamin B1 (Fig. 1d and Supplementary Fig. 2a). CLEM revealed that Emerin-rich MN is composed of NE membranes with extensive distortion and attached chromatin, often with the protrusion of the adjacent ER and cytoplasm to the inside of these structures (Fig. 1e and Supplementary Fig. 2b). Immunogold electron microscopy further confirmed that the presence of Emerin was tightly associated with deformed nuclear membranes (Supplementary Fig. 2b). Since the abundance of Emerin depends on the number of nuclear membranes (which are ER membranes), its density in micronuclei is almost 15 times greater than that in nuclei (Supplementary Fig. 2c). Interestingly, the structure of Emerin-rich MN, with a clear pattern of distorted membranes, resembled that of ruptured micronuclei^1,42^.

### Emerin-rich MN differs in composition from Emerin-NE-level MN

**Fig. 2:**
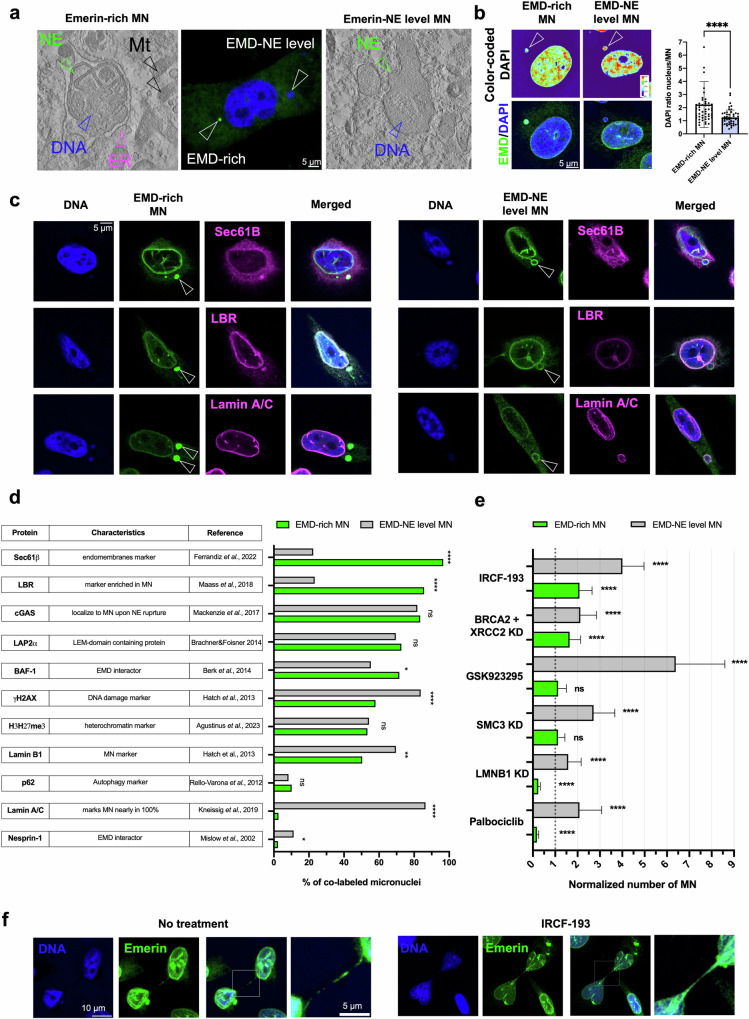
The Emerin expression level can be used to differentiate subtypes of micronuclei. **a** Comparison between Emerin-rich (EMD-rich) MN and Emerin-NE-level (EMD-NE-level) MN using correlative light electron microscopy (CLEM). **b** DAPI signal ratio quantification in Emerin-rich MN (*n* = 51 cells) and Emerin-NE-level MN (*n* = 50 cells). **c** Representative micrographs of staining for Sec61β, Lamin-B receptor (LBR), and Lamin A/C in Emerin-rich MN and Emerin-NE-level MN. Emerin is shown in green, and the other proteins are shown in magenta. **d** Quantification of the percentage of cells with Emerin colabeling in the two MN fractions; a minimum of 250 cells were examined for each condition. **e** Quantification of the Emerin-rich and Emerin-NE-level subtypes of micronuclei after gene silencing and treatment with selected drugs. KD: knockdown; a minimum of 250 cells were examined for each condition. **f** Representative micrographs of Emerin staining in chromatin bridges in cells without treatment and after treatment with the inhibitor IRCF-193. Emerin is shown in green. Proportions quantified using Fisher’s exact test are presented in the bar plot. Scatter plot with bar (mean with SD) and bar plots: Mann–Whitney *U*-test. The error bars indicate the SDs. *p < 0.05; **p < 0.01; ***p < 0.001; ****p < 0.0001; ns, not significant.

To specifically characterize Emerin-rich MN, we investigated their composition using multiple markers. We first classified MN by an Emerin intensity level higher than or at the same level as that of NE, and we then compared their ultrastructures. Interestingly, MN with NE-level Emerin expression did not show the same pattern of NE membrane distortions observed in Emerin-rich MN (Fig. 2a and Supplementary Fig. 3a). Instead, the Emerin-rich MN showed a decreased DAPI signal, a good indicator of the DNA content^43^ (Fig. 2b), and an increased area, probably due to their excessive membrane content (Supplementary Fig. 3b). Micronuclei typically exhibit defective NEs, which can result in enrichment of core NE proteins (Emerin, LAP2α, BAF, Lamin A/C) or noncore proteins, such as nucleoporins, Lamin B1, and the Lamin-B receptor^2,44,45^. Therefore, we tested whether Emerin-rich MN might differ in composition from Emerin-NE-level MN. As expected, due to their high membrane content, Emerin-rich MN more frequently exhibited colabeling of the endomembrane marker Sec61β^46^ and the Emerin interactor BAF-1^47^ (Fig. 2c, d and Supplementary Fig. 3c). In contrast, Emerin-NE-level MN exhibited an increased abundance of the DNA damage marker γH2AX^1^ and decreased expression of the DNA damage marker Lamin B1^48^. cGAS, which localizes to MN upon nuclear envelope rupture^8^, was detected in ~80% of MN in both fractions. Compared with Emerin-NE-level MN, Emerin-rich MN also exhibited more positive staining for the Lamin-B receptor (LBR) and was largely negative for Lamin A/C (Fig. 2c, d). Lamin A/C was previously shown to be detected in nearly 100% of MN, which was also found for Emerin-NE-level MN, as shown in Fig. 2c, d^43^. Surprisingly, another LEM domain-containing protein, LAP2α^49^, was detected at the same frequency in both MN types (Fig. 2c, d). Similarly, lysine trimethylation on histone H3 (H3K27me3), which is often enriched in MN^50^, and the autophagy marker p62^51^ were also detected at the same frequency in both MN types. The Emerin interactor Nesprin-1^52^ was slightly more frequently localized in MN with an Emerin-NE level. Taken together, the collected data suggested that the Emerin level can be used to differentiate MN into two subtypes: the Emerin-NE-level subtype and the Emerin-rich MN subtype. Therefore, we further asked whether the Emerin-rich MN subtype could form as a result of a different event than that leading to the formation of the other subtype of MN.

### Emerin-rich MN originates during chromatin bridge resolution

We tested various known MN-inducing and MN-affecting conditions to determine whether they can promote the formation of one of the subtypes of MN. The depletion of lamin B1, which is known to trigger NE disruption^1^, induced a decrease in the frequency of Emerin-rich MN and an increase in that of Emerin-NE-level MN (Fig. 2e). Interestingly, the formation of only Emerin-NE-level MN increased after exposure to the reported MN-inducing conditions, including knockdown of the cohesin SMC3^53^, treatment with the CENP-E inhibitor GSK923295^46^, and palbociclib incubation^54^ (Fig. 2e and Supplementary Fig. 4a, b). However, conditions such as silencing of genes involved in DNA repair (*BRCA2* and *XRCC2*) and treatment with the topoisomerase II inhibitor IRCF-193^55^ generally increased the frequency of both subtypes of MN (Fig. 2e and Supplementary Fig. 4a, b). As expected, all treatments generally increased the number of both subtypes of MN. However, interestingly, the conditions that more specifically induced the formation of Emerin-rich compared with Emerin-NE-level MN were the ones known to increase the formation of aberrant chromatin bridges upon failed abscission: simultaneous knockdown of *BRCA2* and *XRCC2*^2,9,55,56^ or treatment with the topoisomerase inhibitor IRCF-193 (Fig. 2e and Supplementary Fig. 4a, b). We also observed that Emerin marks chromatin bridges and that Emerin-rich MN are present in cells connected by these structures both without any stimulation and after treatment with ICFR-193 (Fig. 2f). These results therefore suggested that Emerin-rich MN can originate from collapsed or incorrectly resolved chromatin bridges.

**Fig. 3:**
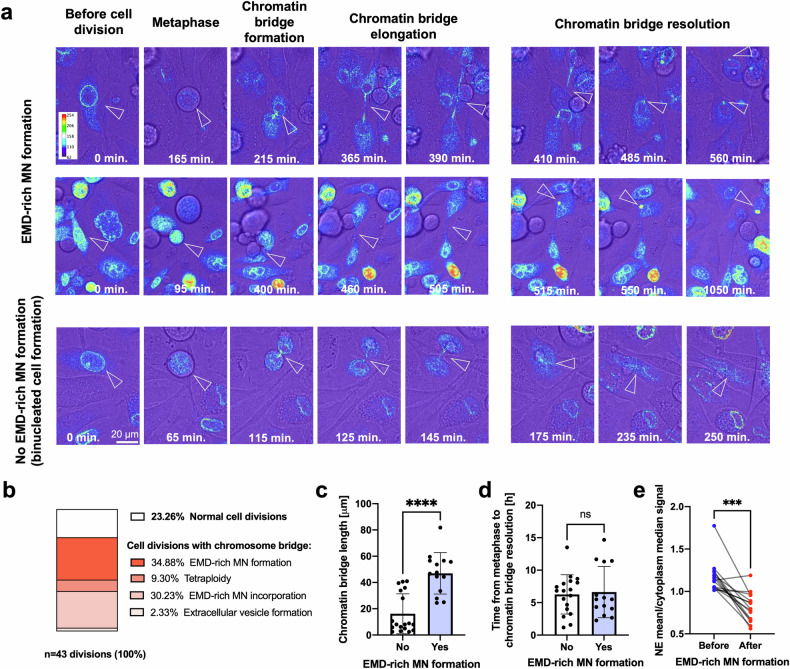
Emerin-rich (EMD-rich) MN is associated with chromatin bridge resolution. **a** Representative micrographs of PC-3 cells transfected with the EGFP-EMD construct showing cell division after IRCF-193 treatment. The arrows indicate chromatin bridges and Emerin-rich MN. **b** Percentages of events associated with cell division in all imaged cells. **c** Quantification of chromatin bridge length during cell division in cells with (*n* = 14) and without (*n* = 18) the formation of Emerin-rich MN. **d** Quantification of chromatin bridge persistence during cell division in cells with (*n* = 14) and without (*n* = 18) the formation of Emerin-rich MN. **e** Quantification of the median NE/cytoplasmic Emerin signal ratio before and after the formation of Emerin-rich MN (*n* = 15). Scatter plot with bar (mean with SD): Mann‒Whitney *U*-test. Paired analysis: Wilcoxon matched-pairs signed-rank test. *p < 0.05; **p < 0.01; ***p < 0.001; ****p < 0.0001; ns, not significant.

To test this hypothesis, we performed a time-lapse analysis of the cell cycle in cells transfected with the EGFP-EMD construct and treated with IRCF-193. Chromatin bridges are aberrant structures that occasionally form in untreated cells and have been shown to persist for 3–20 h^57^; in our case, they lasted for 1.2–14.7 h, with a median of ~6 h. We confirmed the presence of Emerin in these structures; moreover, our data strongly suggested that Emerin-rich MN were formed during the resolution of chromatin bridges (Fig. 3a; Supplementary Movies 1 and 2). Among all the imaged cell divisions, the formation of Emerin-rich MN was the most frequently occurring event associated with chromatin bridge resolution, accounting for ~35% of the investigated events (Fig. 3b). The formation of Emerin-rich MN was associated with the formation and resolution of longer chromatin bridges (Fig. 3c), whereas shorter chromatin bridges often led to binucleated cell formation (Fig. 3a, b and Supplementary Movie 3). We did not observe a correlation between the formation of Emerin-rich MN and the duration of chromatin bridge persistence (Fig. 3d). Interestingly, we also observed that the presence of Emerin-rich MN completely changed the NE-to-cytoplasm ratio of this protein (Fig. 3e), indicating that Emerin could be pauperized from NEs in favor of MN.

### Emerin-rich MN causes Emerin pauperization at the NE

**Fig. 4:**
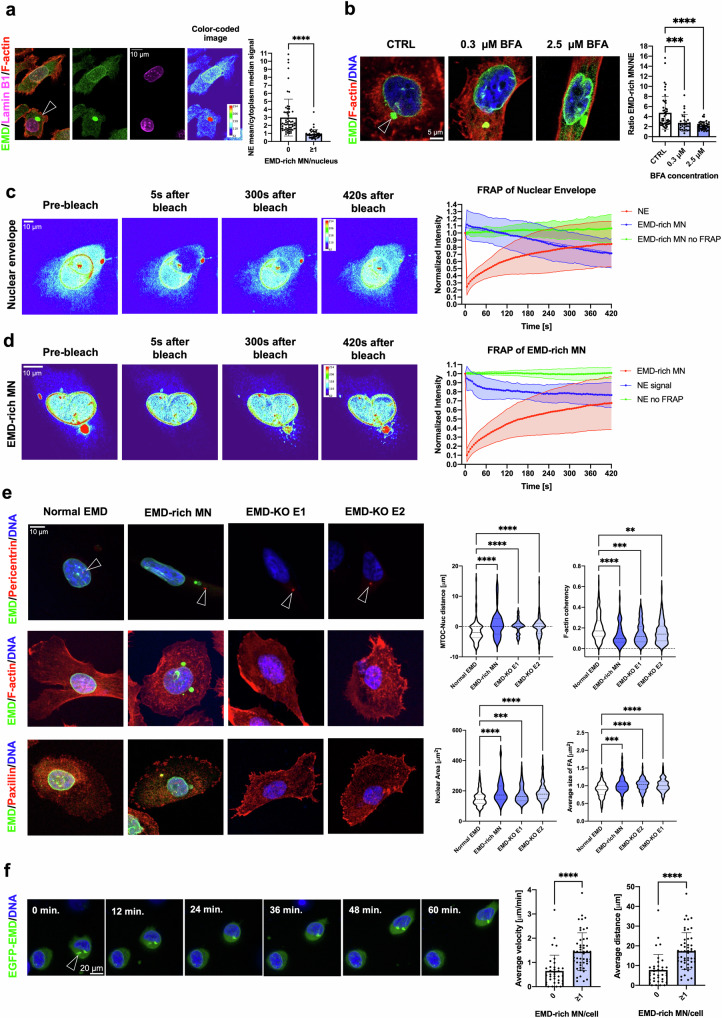
The formation of Emerin-rich (EMD-rich) MN can induce Emerin pauperization at the NE and lead to the reproduction of the Emerin loss phenotype. **a** Quantification of the median NE/cytoplasmic Emerin signal ratio in fixed PC-3 cells with (*n* = 51) and without Emerin-rich MN (*n* = 62). **b** Quantification of the Emerin intensity signal ratio between Emerin-rich MN and the NE in the CTRL (*n* = 48) and Brefeldin A treatment (0.3 μM (*n* = 30) and 2.5 μM (*n* = 40)) groups. **c** iFRAP analysis of PC-3 cells transfected with the EGFP-EMD construct. The region of the nuclear envelope was subjected to photobleaching (*n* = 23 cells), and the control cells were not subjected to photobleaching (*n* = 20 cells). **d** iFRAP analysis of PC-3 cells transfected with the EGFP-EMD construct. The region of the Emerin-rich MN was subjected to photobleaching (*n* = 15 cells), and the control cells were not subjected to photobleaching (*n* = 20 cells). **e** Quantification of selected parameters in PC-3 cells without Emerin-rich MN, with Emerin-rich MN, with EMD-KO E1 and with EMD-KO E2: nucleus-MTOC distance (*n* = 123, *n* = 52, *n* = 79, *n* = 106, respectively), F-actin coherency (*n* = 145, *n* = 66, *n* = 136, *n* = 167, respectively), nuclear area (*n* = 123, *n* = 52, *n* = 79, *n* = 106, respectively), and focal adhesion size (*n* = 221, *n* = 73, *n* = 130, *n* = 141, respectively). **f** Assessment of the migratory potential of PC-3 cells transfected with EGFP-EMD with (*n* = 50) and without (*n* = 36) Emerin-rich MN. The average cell velocity and distance were calculated from individual paths. Scatter plot with bar (mean with SD): Mann–Whitney *U*-test. Violin plot: minimum to maximum values, specifically, the first quartile, median, and third quartile values. For comparisons among more than two groups, the Kruskal‒Wallis test was used. *p < 0.05; **p < 0.01; ***p < 0.001; ****p < 0.0001; ns, not significant.

To further investigate how the presence of Emerin-rich MN in the cytoplasm impacts Emerin localization at the NE, we quantified the intensity ratio of Emerin at the NE and in the cytoplasm by immunofluorescence staining. Cells with Emerin-rich MN showed significant pauperization of this protein from the NE (Fig. 4a). In general, decreased Emerin expression during malignant transformation is associated with the nuclear structural defects required for the increased migratory and invasive capacities of cancer cells, leading to increased metastatic potential and unfavorable prognosis^15,40,41^. Emerin, in addition to being located at the inner and outer nuclear membrane, is also present at the ER membrane^11,58^, an observation that could explain the accumulation of membranes and Emerin in the Emerin-rich MN. It was previously shown that Emerin can be rapidly cleared from the nuclear membrane by vesicular transport^58^. We also observed that treatment with brefeldin A (BFA), an agent that inhibits intracellular trafficking, abolished Emerin pauperization at the NE (Fig. 4b). To further determine whether there is a physical interconnection between Emerin-rich MN and the NE, we performed iFRAP (inverse fluorescence recovery after photobleaching). Emerin was photobleached from the NE in EGFP-EMD-transfected cells, and this event was associated with the decay of the fluorescence signal from Emerin-rich micronuclei (Fig. 4c and Supplementary Movie 4). Similarly, when the Emerin-rich MN was photobleached, the EGFP-EMD signal intensity decreased at the NE (Fig. 4d and Supplementary Movie 5). Additionally, the pattern of Emerin trafficking might resemble that of vesicle-based trafficking rather than that of diffusion^29^. The collected data showed that Emerin can shuffle between these two compartments, which could be a mechanism to modulate the presence of Emerin in both MN and the NE. As we confirmed that NE pauperization can occur, we further aimed to test whether NE pauperization can also be translated to a cellular phenotype.

### The presence of Emerin-rich MN mimics the phenotype of Emerin loss at the cellular level

Emerin loss is associated with a characteristic cellular phenotype that includes an increased distance between the microtubule-organizing center (MTOC) and the nucleus^11,59,60^, a more random F-actin fiber organization due to altered actin dynamics^59^, alterations in the size of focal adhesions^11^, an increased nuclear area and an irregular nuclear shape^61^, and increased migratory and invasive potential^11,15,40^. Decreased and deregulated expression of Emerin has also been reported to increase the metastatic potential of PCa cells^15^. To determine whether the presence of Emerin-rich MN could mimic the phenotype associated with loss of Emerin, we compared some of the phenotypic changes in cells without MN, cells with Emerin-rich MN and EMD-knockout (EMD-KO) cells (Supplementary Fig. 5a). Indeed, cells containing Emerin-rich MN phenotypically resembled cells with Emerin deficiency (Fig. 4e), suggesting that the accumulation of Emerin in the cytoplasmic compartment can lead to altered cellular properties. Importantly, cells with an Emerin deficiency phenotype caused by the presence of Emerin-rich MN or EMD-KO had larger focal adhesions, suggesting a more migratory phenotype^62,63^. To functionally test whether cells with Emerin-rich MN are characterized by a migratory phenotype, we first performed a 2D random migration analysis. We observed that cells with Emerin-rich structures tend to move faster and along longer pathways (Fig. 4f and Supplementary Movie 6), as previously observed in Emerin-deficient cells^11^. Therefore, we further investigated cells with Emerin-rich MN and Emerin pauperization in the context of PCa.

### The transcriptomic signature of pauperized Emerin in PCa

**Fig. 5:**
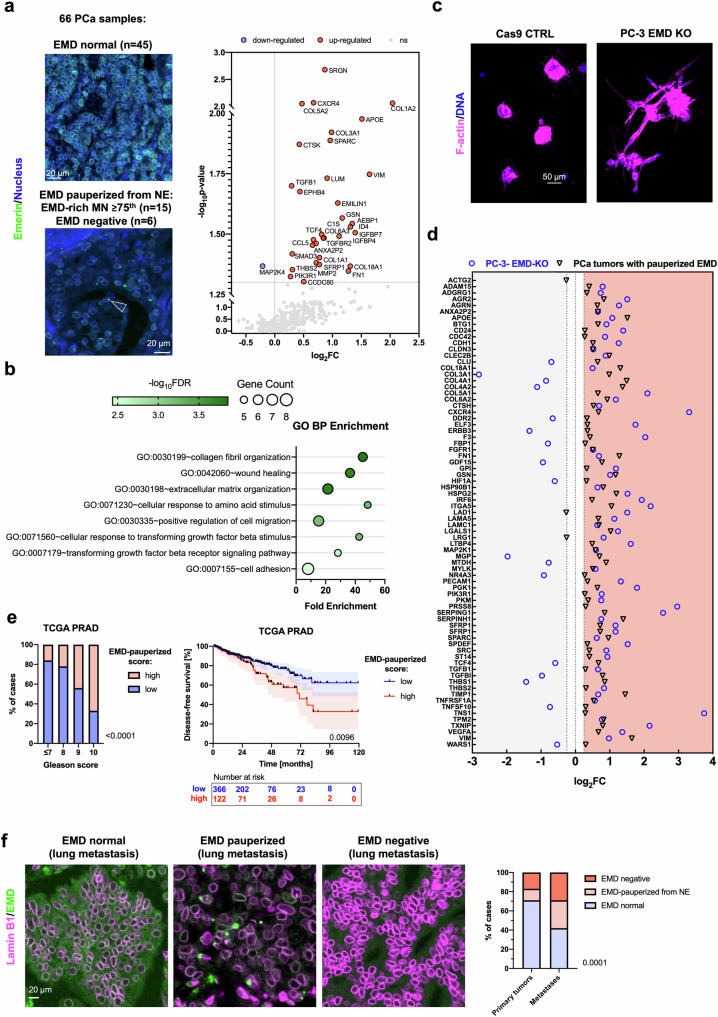
The transcriptomic signature of PCa cells with Emerin pauperization. **a** nCounter analysis of prostate tumors (*n* = 66) divided into 2 groups based on the pattern of Emerin expression: normal NE expression of Emerin and Emerin pauperization at the NE (presence of Emerin-rich [EMD-rich] MN or negativity for Emerin). The scatter plot shows the differentially expressed genes. **b** Gene Ontology analysis of upregulated genes in tumors with Emerin pauperization at the NE. **c** Growth of PC-3 cells with EMD-KO cells under 3D culture conditions. **d** Forest plot of 75 genes common to both datasets—the nCounter NanoString dataset for prostate tumors and the RNA sequencing dataset for PC-3 cells grown under 3D culture conditions. To facilitate visual representation, the point representing the *ACTG2* gene in the EMD-KO group is not shown in the graph (log_2_FC = 5.861). **e** Analysis of Emerin pauperization in the TCGA PRAD dataset and its association with the Gleason score and disease-free survival (*n* = 488). **f** Distribution of Emerin phenotypes (normal, pauperized, and negative) in prostate primary tumors (*n* = 278 samples, corresponding to 117 patients) and prostate cancer metastatic lesions (*n* = 28 samples, corresponding to 14 patients). Bar plot: chi-square test. Kaplan–Meier curve: log–rank test; the error bars indicate the 95% CIs. *p* < 0.05; **p < 0.01; ***p < 0.001; ****p < 0.0001; ns, not significant.

To verify the signatures of Emerin-rich MN and loss of Emerin in prostate tumors, the transcriptomic profiles of tumors were analyzed using the nCounter PanCancer Progression Panel. Based on the pattern of immunofluorescence staining for Emerin, tumors were divided into two groups: one with a normal pattern of Emerin staining at the NE and another with a staining pattern associated with pauperization of Emerin from the NE (presence of Emerin-rich MN or Emerin loss in cancer cells, Fig. 5a). The expression of 33 genes (Supplementary Table 3), including those encoding proteins involved in prostate cancer migration and invasion, such as vimentin^64^ and SPARC^65^, or invasion, such as MMP2^66^, was upregulated in tumors with Emerin pauperization the Nat E, as presented in Fig. 5a and Supplementary Fig. 5b. Gene Ontology analysis revealed that among the enriched processes in tumors with pauperized Emerin were cell adhesion, positive regulation of cell migration and response to TGFβ (Fig. 5b). When we grew EMD-KO PC-3 cells under 3D culture conditions, we observed that they had a more invasive morphology than those formed from control cells—the EMD-KO 3D spheroids were larger, more branched, and less circular than the 3D spheroids formed from control cells (Fig. 5c and Supplementary Fig. 5c). We further analyzed these spheroids using RNA sequencing. Then, we verified whether the transcriptomic signature of EMD-KO PC-3 cells grown under 3D conditions shares any similarities with that of PCa cells with pauperized Emerin. Indeed, there was a common pattern of gene upregulation in both datasets (Fig. 5d). The top 9 upregulated genes in both datasets were *CXCR4*, *APOE*, *SPARC*, *VIM*, *GSN*, *ANXA2P2*, *SFRP1*, *COL18A1*, and *FN1* (Fig. 5d and Supplementary Table 4).

### The Emerin pauperization transcriptomic score predicts a shorter time to progression in the TCGA dataset and the associated transcriptomic signature is more frequently found in metastatic PCa

We analyzed the TCGA dataset to evaluate the potential adverse impact of Emerin pauperization on prostate cancer prognosis. We computed the transcriptomic score including the top 9 upregulated genes in prostate tumor tissues with Emerin pauperization in the NE and EMD-KO experiments (*CXCR4, APOE, SPARC, VIM, GSN, ANXA2P2, SFRP1, COL18A1, and FN1*), and compared it with the patients’ clinical characteristics and disease-free survival (DFS) time, defined as the time to disease recurrence or progression. The Emerin pauperization score increased with increasing Gleason score, and importantly, a high score was linked to a shorter DFS time (HR: 1.75; 95% CI: 1.14–2.69; *p*-value: 0.0106, as shown in Fig. 5e). The transcriptomic data obtained from tumor samples collected from 66 PCa patients and from 3D-cultured PC-3 cell spheroids were largely consistent and together suggested that cells with Emerin pauperization exhibit increased migratory and invasive potential, which could subsequently increase their metastatic potential and be a marker of poor prognosis in PCa patients. To further test the relationship between Emerin pauperization at the NE and unfavorable prognosis, we measured Emerin pauperization in samples of 28 metastatic prostate cancer lesions (from 14 PCa patients, Supplementary Table 5). We found that the Emerin pauperization phenotype was twice as frequently detected in metastatic samples than in unmatched primary prostate tumors (Fig. 5f and Supplementary Table 6).

### The Emerin pauperization phenotype is associated with a collagen-rich microenvironment, which is associated with increased invasive potential

**Fig. 6:**
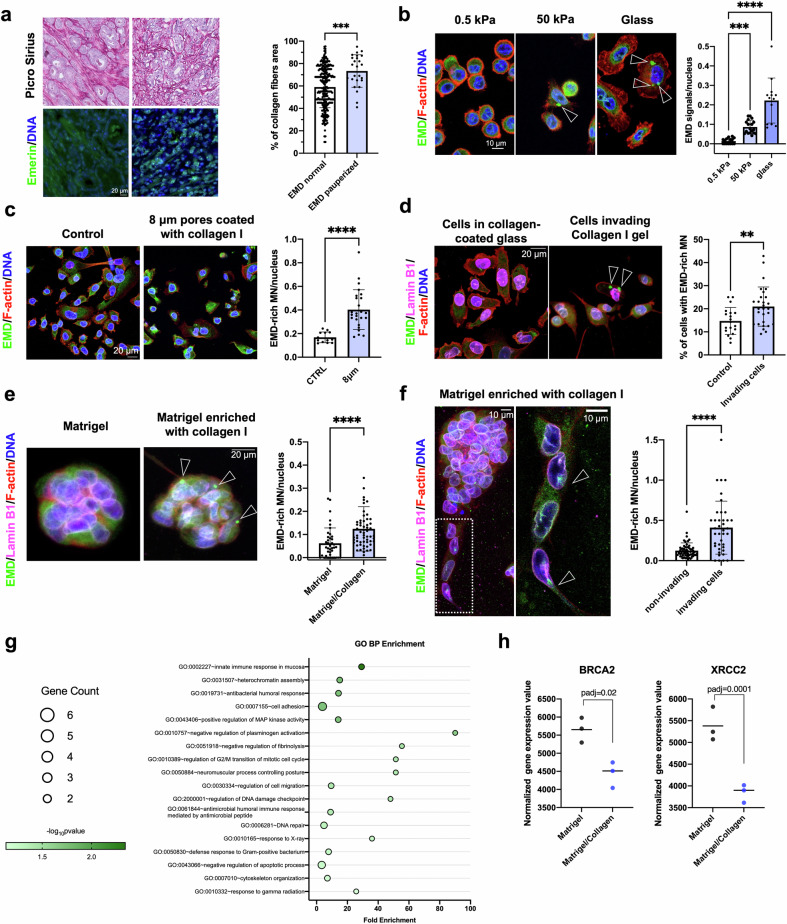
Cells with Emerin-rich (EMD-rich) MN exhibit increased migratory and invasive potential. **a** Analysis of the percentage area of collagen fibers in primary tumors with a normal Emerin expression pattern (*n* = 256) and Emerin pauperization (*n* = 25) using Picrosirius Red staining. **b** Quantification of Emerin-rich MN in cells grown under different conditions: 0.5 kPa collagen-coated hydrogel (*n* = 15 fields of view), 50 kPa collagen-coated hydrogel (*n* = 18 fields of view), and collagen-coated glass (*n* = 13 fields of view). For all quantifications, >250 cells were used. **c** Quantification of Emerin-rich MN/nuclei in PC-3 cells cultured under control conditions (*n* = 13 fields of view with >250 cells) and in cells that passed through 8 µm pores (*n* = 25 fields of view with >250 cells). **d** Quantification of Emerin-rich MN/nuclei in PC-3 cells cultured under control conditions (*n* = 19 fields of view with >250 cells) and in cells that invaded a collagen layer (*n* = 28 fields of view with >250 cells) in a collagen invasion assay. **e** Assessment of Emerin-rich MN in spheroids cultured in Matrigel alone (*n* = 39 spheroids) and in collagen-supplemented Matrigel (*n* = 58 spheroids). **f** Assessment of Emerin-rich MN/nuclei in noninvading (*n* = 55 spheroids) and invading (*n* = 43 spheroids) spheroids formed from PC-3 cells in 3D culture. **g** Gene Ontology analysis of downregulated genes in spheroids grown in a collagen-enriched microenvironment. **h** Downregulation of two genes associated with DNA repair, *BRCA2* and *XRCC2*, in a collagen-enriched microenvironment; 3 independent experiments. Scatter plot with bar (mean with SDSD): Mann‒Whitney *U*-test. *p < 0.05; **p < 0.01; ***p < 0.001; ****p < 0.0001; ns, not significant.

**Fig. 7:**
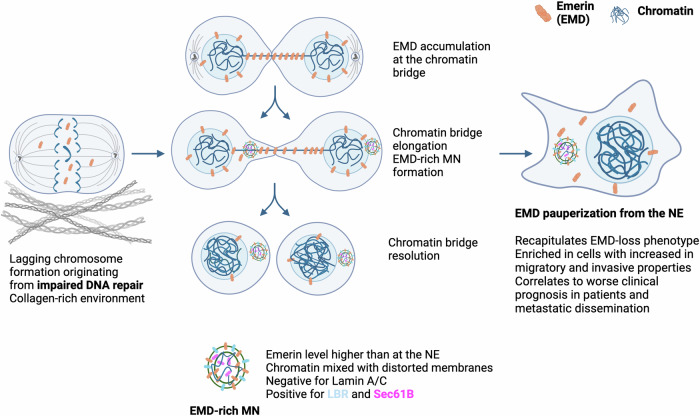
Proposed mechanism for the formation of Emerin-rich (EMD-rich) MN during chromatin bridge resolution, followed by Emerin mislocalization from the nuclear envelope and phenotypic changes in cells.

Interestingly, in the TCGA dataset, the Emerin pauperization phenotype was also positively correlated with *COL1A1* expression (Supplementary Fig. 5d). To experimentally test this finding, we evaluated the percentage area of collagen fibers in primary prostate tumors using Picrosirius Red staining (Fig. 6a). The amount of Picrosirius Red staining correlated with *COL1A1* mRNA expression in these tumors (Supplementary Fig. 5e). We found that the Emerin pauperization phenotype was associated with an increased area of collagen fibers (Fig. 6a). Since the major factor contributing to altered extracellular stiffness is increased deposition of type I collagen^67,68^, we then tested whether the number of Emerin-rich MN increases with increasing substrate stiffness. By culturing cells on hydrogels of different stiffnesses coated with collagen I, we observed an increase in the number of Emerin-rich MN on the stiffer hydrogel (Fig. 6b). Similarly, in the transwell migration assay, cells that invaded through the membrane (coated with collagen I) had significantly more Emerin-rich MN than did the noninvading ones (Fig. 6c), and this pattern was also observed in the collagen invasion assay (Fig. 6d). The frequency of cells in spheroids with Emerin-rich MN was much greater in the collagen-rich microenvironment (Fig. 6e), especially for invading cells compared to the noninvading ones (Fig. 6f). To investigate the potential molecular candidates responsible for the formation of Emerin-rich MN, we performed RNA sequencing of cells grown in these two microenvironments (Matrigel alone and Matrigel supplemented with collagen I). Gene Ontology analysis revealed that among the depleted terms in the collagen-enriched microenvironment were the ones related to DNA repair, such as response to X-ray and gamma radiation (Fig. 6g). Notably, *BRCA2* and *XRCC2*, whose simultaneous knockdown led to increased formation of Emerin-rich MN (Fig. 2e), were both downregulated in the collagen-enriched gel (Fig. 6h). This evidence further supports our hypothesis that in a collagen-rich microenvironment, impairment of DNA repair processes could lead to increased chromatin bridge formation followed by the formation of Emerin-rich MN and subsequent Emerin pauperization at the NE (Fig. 7).

Taken together, our data suggest that the formation of Emerin-rich MN, which results in functional pauperization of Emerin at the nuclear envelope, causes a specific phenotypic switch in prostate cancer cells, ultimately resulting in more invasive behavior strongly linked to a worse prognosis.

## Discussion

In the present study, we showed that the presence of micronuclei could induce phenotypic and functional changes in cells and increase their migratory and invasive potential. Mechanistically, we proposed that MN formation could cause pauperization of the Emerin protein at the NE.

Emerin mislocalization was previously reported in 80 PCa cases, where it discriminated tumor tissue from nontumor tissue and was correlated with disease progression^15^. We confirmed this observation in a slightly larger cohort of patients with an increased risk of metastasis (defined as intermediate- to high-risk PCa by the D’Amico classification) and showed that we can even stratify patients by the frequency of Emerin-rich MN, which correlates with the Gleason score, the time to biochemical recurrence and the occurrence of metastasis after radical prostatectomy. By combining confocal fluorescence and electron microscopy, we were able to investigate the ultrastructure and composition of these structures. We also observed that the abundance of Emerin can be used to differentiate subtypes of MN, but the classification scheme was not consistent with the previously proposed classification of “core” and “noncore” protein expression^2^ or based on the presence of membrane rupture^1,8^, suggesting the different origins of the subtypes. Essentially, we found that the ultrastructure and composition of Emerin-rich MN differ from those of Emerin-NE-level MN, suggesting that the two subtypes could be formed via different mechanisms.

Emerin has previously been found to assemble on lagging chromosomes or chromatin bridges, with a lack of LBR on these structures^2^. Consistent with this observation, here, we showed that Emerin-rich micronuclei likely form during chromatin bridge collapse. As previously observed, chromosome bridge formation predisposes cells to micronucleation^55,69^. Moreover, we observed that Emerin-rich MN formed more often in longer chromatin bridges, regardless of their lifetime, and it has already been proposed that cellular mechanical forces can stretch chromatin bridges. We then hypothesized that long chromatin bridges could drive severe accumulation of NE membranes, which, upon the abrupt resolution of the bridge, leads to the formation of micronuclei with the characteristic membrane distortions observed in Emerin-rich MN. The resolution of these long chromatin bridges, which induces the formation of Emerin-rich MN, ultimately causes Emerin pauperization at the NE. Indeed, Emerin can shuffle between Emerin-rich MN and the NE, and the abundant membranes in Emerin-rich MN act as a sink for Emerin and cause its pauperization at the NE. This mechanism could allow the modulation of Emerin localization at the NE without affecting *EMD* gene expression. The Emerin level at the NE has regulatory effects on important cellular processes, such as cell motility^70^ and cell polarization^10,11^. Moreover, Emerin loss is associated with altered nuclear envelope elasticity, which contributes to increased nuclear fragility^71^. As Emerin’s main interacting protein, BAF^47^ could play a role in NE repair and cGAS-mediated signaling^72^, and Emerin could also be involved in these processes either directly or indirectly. Its interaction with lamin A^73^ and actin filaments^59^ can affect nuclear blebbing^74^, the mechanical response of nuclei^75^, and chromatin architecture^76^; consequently, it might also influence nuclear structure. Generally, decreased Emerin expression during malignant transformation is associated with nuclear structural defects, increased cancer cell migration and invasion, increased metastatic potential, and unfavorable prognosis^15,40,41^. Importantly, cells positive for Emerin-rich MN partially phenocopied EMD-KO cells, and they displayed larger focal adhesions, suggesting a greater migratory capacity^62,63^.

Previously, Emerin mislocalization has been detected in PCa and linked to metastatic potential. Circulating tumor cells from prostate cancer patients were shown to have Emerin mislocalization, and the DU145 prostate cancer cells with *EMD* silencing formed widespread metastases more efficiently in a mouse model^15^. Our data from tumor samples collected from PCa patients and PC-3 cell spheroids formed in 3D culture suggest that cells with Emerin pauperization share a common transcriptomic signature. Most genes involved in cell migration and invasion were upregulated, and many of them were previously linked with prostate cancer progression^64–66,77^. Moreover, the set of genes upregulated in cells with Emerin pauperization could indicate a worse prognosis in patients. Additionally, the Emerin- pauperization phenotype was more prevalent in metastatic lesions than in primary tumors from patients with PCa, further characterizing cells with invasive potential. To our knowledge, this is the first study to report such data.

As the Emerin pauperization phenotype was associated with increased *COL1A1* expression, we were also able to confirm the enrichment of cells with Emerin-rich MN in the population of the most migratory and invasive cells using functional assays in the context of a collagen-rich microenvironment. Our observation of an amplified epithelial–mesenchymal plasticity phenotype could indicate increased tissue fluidification^78^. Such mechanically driven transcriptional reprogramming is associated with cell state alterations accompanied by the acquisition of malignant traits, including epithelial-to-mesenchymal plasticity phenotypes, in a collagen-rich microenvironment and could explain the poorer patient prognosis. Interestingly, *BRCA2* and *XRCC2*, two genes whose simultaneous knockdown led to increased formation of Emerin-rich MN, were both downregulated in a collagen-rich microenvironment. This finding suggested that an increase in collagen content could lead to impaired DNA damage repair, chromosome bridge formation, and Emerin pauperization at the NE, which could lead to increased invasive potential.

Emerin pauperization at the NE might participate in the complex series of events that drive prostate tumor progression. At the same time, chromatin bridge collapse can lead to the formation of Emerin-rich MN, inducing Emerin pauperization at the NE and DNA damage, resulting in extreme genomic complexity and continuing evolution of subclonal heterogeneity^55^. In turn, downstream of chromosome bridge formation, the increasing burden of DNA breakage can easily exceed the capacity to stabilize broken chromosome ends. Therefore, complex genome evolution with subclonal heterogeneity is a virtually inevitable consequence of chromosome bridge formation, which itself is a common result of cell division defects during tumorigenesis. Both genomic instability and deficient DNA repair mechanisms have been reported in PCa, where they correlate with both a poor prognosis and a migratory phenotype^18–20,79^. Here, we demonstrated that the presence of Emerin-rich MN could serve as a predictor of progression in patients with PCa. However, such data should be verified in a larger cohort.

Taken together, via a variety of imaging and molecular methods, we were able to show that chromosome bridge resolution can lead to Emerin accumulation and the formation of Emerin-rich MN. These MN are negative for Lamin A/C and positive for LBR and Sec61β. As a protein sink, these MN cause Emerin pauperization at the NE. This, in turn, is functionally linked to the migratory and invasive potential of cells and can translate to poor prognosis in patients. Considering the ubiquitous expression of Emerin across tissues and various cancers^41,80^, the pathogenic effect of Emerin pauperization could also extend to other malignant diseases.

## Supplementary information

Supplementary Information

Supplementary Movie 1

Supplementary Movie 2

Supplementary Movie 3

Supplementary Movie 4

Supplementary Movie 5

Supplementary Movie 6

## Data Availability

The RNA-seq datasets generated during the current study are available in the NCBI Sequence Read Archive (SRA) under accession number PRJNA1113153. The datasets used and/or analyzed in this article are included within the article and the additional files. All data are available in the main text or the supplementary materials. Please contact the corresponding author for data requests.
