# Supplementary material for: Emerin mislocalization during chromatin bridge resolution can drive prostate cancer cell invasiveness in a collagen-rich microenvironment
Source: Exp Mol Med. 2024 Sep 2;56(9):2016–32. doi: 10.1038/s12276-024-01308-w (PMC11446916; doi:10.1038/s12276-024-01308-w)
Supplement: Supplementary file 1 — Supplementary Information [file 12276_2024_1308_MOESM1_ESM.pdf]

Supplementary Information

Supplementary Material and Methods

Supplementary Tables 1 to 8

Supplementary Figures 1 to 5

Caption to Supplementary Movies 1-6

Supplementary Material and Methods

RNA expression in PC-3 cell line. Data quality was checked using FastQC 0.11.9 [1] and MultiQC 1.11 [2] reporting tools. After quality control, raw reads were trimmed using Trimmomatic 0.39 [3] and then aligned to the human genome GRCh38.p10 using STAR 2.7.10 [4]. Mapped reads were counted across genomic features using featureCounts 2.0.3 [5]. Read counts were normalized and then subjected to differential analysis using DESeq2 1.360.0 [6]. The absolute value of log2 fold change ≥ (0.5) and adjusted p-value < 0.05 were used as criteria to identify differentially expressed genes.

Membrane measurement of MN and NE. Since correlative light electron microscopy [7] deals with tangential sections of cells, we used the Cavalieri method adapted to micronuclei cut tangentially [7-11]. We used the 200 nm tomography sections for our analysis. In each serial 200 nm slice, where the nucleus or micronucleus (MN) was visible, using the RADIUS (ThermoFisher) program in cells where MN was detected, we measured the area of the micronucleus slice, the length of the perimeter of its nuclear envelope and the length of the membranes of the invaginations of the nuclear envelope on each serial slice. In neighboring cells where MN was not found, we measured the area of the section of the nucleus, the length of the perimeter of the nuclear envelope and the length of the membranes of the invaginations of the nuclear envelope. Additionally, for each nucleus and each micronucleus, we calculated the average ratio of the surface area to the length of the nuclear membranes (the sum of the perimeter and invaginations). The control nucleus was considered to be in a cell that was located next to a cell with a micronucleus. In total, 5 randomly selected nuclei of neighboring cells and 15 MNs were measured. Then we calculated the ratio between the surface area and the length of nuclear double membranes for all slices of a given nucleus and MN. Then the average ratio was calculated. We did not take the nucleus of the cell where MN was detected into our calculations, since part of the DNA went into MN there, which violated the ratio of the nuclear shell to the volume of the nucleus. We considered the resulting average ratio as a single statistical value (variant) for a particular core or microkernel. That is, there are 15 average values for 15 microkernels and 5 average values for neighboring cores (Supplementary Fig. 2c).

References

1 Andrews S *et al.* in *1:1* (Babraham Bioinf, 2015).

2 Ewels, P., Magnusson, M., Lundin, S. & Käller, M. MultiQC: summarize analysis results for multiple tools and samples in a single report. *Bioinformatics* **32**, 3047-3048 (2016). https://doi.org/10.1093/bioinformatics/btw354

3 Bolger, A. M., Lohse, M. & Usadel, B. Trimmomatic: a flexible trimmer for Illumina sequence data. *Bioinformatics* **30**, 2114-2120 (2014). https://doi.org/10.1093/bioinformatics/btu170

4 Dobin, A. *et al.* STAR: ultrafast universal RNA-seq aligner. *Bioinformatics* **29**, 15-21 (2013). https://doi.org/10.1093/bioinformatics/bts635

5 Liao, Y., Smyth, G. K. & Shi, W. featureCounts: an efficient general purpose program for assigning sequence reads to genomic features. *Bioinformatics* **30**, 923-930 (2014). https://doi.org/10.1093/bioinformatics/btt656

6 Love, M. I., Huber, W. & Anders, S. Moderated estimation of fold change and dispersion for RNA-seq data with DESeq2. *Genome Biol* **15**, 550 (2014). https://doi.org/10.1186/s13059-014-0550-8

7 Beznoussenko, G. V. & Mironov, A. A. Correlative video-light-electron microscopy of mobile organelles. *Methods Mol Biol* **1270**, 321-346 (2015). https://doi.org/10.1007/978-1-4939-2309-0_23

8 Mironov, A. A. Estimation of subcellular organelle volume from ultrathin sections through centrioles with a discretized version of the vertical rotator. *J Microsc* **192**, 29-36 (1998). https://doi.org/10.1046/j.1365-2818.1998.00392.x

9 Mironov, A. A., Beznusenko, G. V., Sesorova, I. S. & Banin, V. V. [How to measure structures, or new stereology: III. Stereology and electron microscopy]. *Morfologiia* **129**, 72-75 (2006).

10 Beznoussenko, G. V. *et al.* Trans-membrane area asymmetry controls the shape of cellular organelles. *Int J Mol Sci* **16**, 5299-5333 (2015). https://doi.org/10.3390/ijms16035299

11 Beznoussenko, G. V., Ragnini-Wilson, A., Wilson, C. & Mironov, A. A. Three-dimensional and immune electron microscopic analysis of the secretory pathway in Saccharomyces cerevisiae. *Histochem Cell Biol* **146**, 515-527 (2016). https://doi.org/10.1007/s00418-016-1483-y

Supplementary Table 1 Clinical and pathological parameters of prostate cancer cohort.

Clinical and pathological parameters		n	%
**Age**	≤median (65)	52	44.4
	>median (65)	65	55.6
	Total	117	
**T status**	pT2	62	53.0
	pT3	55	47.0
	Total	117	
**N status**	N0	108	94.7
	N1	6	5.3
	Total	114	
**Gleason score**	7	82	73.9
	≥8	29	26.1
	Total	111	
**Pre-operative PSA**	<10 ng/ml	80	68.4
	≥10 ng/ml	37	31.6
	Total	117	
**Biochemical recurrence**	No	71	65.1
	Yes	38	34.9
	Total	109	
**Metastasis**	No	110	94.0
	Yes	7	6.0
	Total	117	

Supplementary Table 2 Uni- and multivariate analysis of association between Emerin and clinicopathological parameters and Biochemical Recurrence in prostate cancer cohort.

	univariate				multivariate			
	**HR**	**CI**	**CI**	**pvalue**	**HR**	**CI**	**CI**	**pvalue**
**Emerin-rich MN <75th vs. ≥75th percentile**	3.13	1.64	5.98	0.0006	2.98	1.53	5.84	0.0014
**Age ≤65 vs. >65**	0.93	0.49	1.75	0.8160	-	-	-	-
**Pre-operative PSA <10 vs. ≥ 10 [ng/ml]**	1.42	0.74	2.73	0.2880	-	-	-	-
**pT2 vs. pT3**	2.08	1.06	4.08	0.0342	1.68	0.80	3.52	0.1707
**pN0 vs. pN1**	3.31	1.15	9.51	0.0261	2.70	0.88	8.27	0.0829
**Gleason score 7 vs. ≥8**	2.17	1.12	4.20	0.0217	2.21	1.11	4.39	0.0238

Supplementary Table 3 Differentially expressed genes among tumors with Emerin-rich MN or Emerin pauperized from NE.

Symbol	Entrez	log2FC	p-val
SRGN	5552	0.87	0.002
CXCR4	7852	0.67	0.009
COL1A2	1278	2.04	0.009
COL5A2	1290	0.47	0.009
APOE	348	1.51	0.011
COL3A1	1281	0.98	0.012
SPARC	6678	0.96	0.013
CTSK	1513	0.43	0.013
VIM	7431	1.64	0.018
LUM	4060	0.91	0.019
TGFB1	7040	0.29	0.020
EPHB4	2050	0.43	0.021
EMILIN1	11117	1.09	0.024
GSN	2934	1.17	0.027
AEBP1	165	1.34	0.029
ID4	3400	1.31	0.029
IGFBP7	3490	1.39	0.031
TGFBR2	7048	0.81	0.032
IGFBP4	3487	1.12	0.032
COL6A3	1293	0.85	0.033
C1S	716	0.84	0.033
TCF4	6925	0.67	0.033
CCL5	6352	0.72	0.035
ANXA2P2	304	0.66	0.035
SMAD3	4088	0.30	0.038
COL1A1	1277	0.78	0.040
SFRP1	6422	0.72	0.042
MMP2	4313	0.77	0.042
MAP2K4	6416	-0.21	0.043
COL18A1	80781	1.31	0.043
THBS2	7058	0.31	0.044
FN1	2335	1.28	0.045
PIK3R1	5295	0.27	0.047
CCDC80	151887	0.50	0.050

Supplementary Table 4 The top 9 up-regulated genes in both datasets – RNA sequencing of PC-3 (control vs. EMD-KO) and Nanostring analysis of tumors with Emerin-rich MN or Emerin pauperized from NE vs normal Emerin.

external_gene_name	log2FC RNAseq	p-val RNAseq	log2FC Nanostring	p-val Nanostring
CXCR4	3.31	1.50127E-08	0.67	0.0085459
APOE	1.07	0.00182509	1.51	0.0105515
SPARC	0.62	0.041990448	0.96	0.0129548
VIM	0.98	1.36492E-09	1.64	0.0178852
GSN	1.01	7.49137E-12	1.17	0.0271238
ANXA2P2	0.66	0.001324555	0.66	0.0351556
SFRP1	1.17	0.000457857	0.72	0.0415546
COL18A1	0.50	2.4982E-05	1.31	0.0429317
FN1	0.69	7.89609E-05	1.28	0.0449681

Supplementary Table 5 Prostate cancer metastasis – sample characteristics.

Number	TMA	Patient	Organ	Type
1	PCMET2	G-01-JA	brain	metastasectomy
2	PCMET2	G-01-JA	brain	metastasectomy
3	PCMET2	G-02-MJ	liver	biopsy
4	PCMET2	G-02-MJ	liver	biopsy
5	PCMET2	G-03-NW	lung	metastasectomy
6	PCMET2	G-03-NW	lung	metastasectomy
7	PCMET2	G-04-PJ	distant lymph node	metastasectomy
8	PCMET2	G-04-PJ	distant lymph node	metastasectomy
9	PCMET2	G-05-PS	bone	biopsy
10	PCMET2	G-05-PS	bone	biopsy
11	PCMET2	G-06-RE	lung	metastasectomy
12	PCMET2	G-06-RE	lung	metastasectomy
13	PCMET2	G-07-SE	bone	biopsy
14	PCMET2	G-07-SE	bone	biopsy
15	PCMET2	G-08-WW	lung	metastasectomy
16	PCMET2	G-08-WW	lung	metastasectomy
17	PCMET1	G-10-BK	brain	metastasectomy
18	PCMET1	G-10-BK	brain	metastasectomy
19	PCMET1	G-11-JJ	lung	biopsy
20	PCMET1	G-12-JM	distant lymph node	metastasectomy
21	PCMET1	G-12-JM	distant lymph node	metastasectomy
22	PCMET1	G-14-MJ	distant lymph node	biopsy
23	PCMET1	G-14-MJ	distant lymph node	biopsy
24	PCMET1	G-15-PZ	penis	metastasectomy
25	PCMET1	G-15-PZ	penis	metastasectomy
26	PCMET1	G-15-PZ	urethra	metastasectomy
27	PCMET1	G-16-SJ	lung	metastasectomy
28	PCMET1	G-16-SJ	lung	metastasectomy

Supplementary Table 6 Emerin status in metastatic samples form PCa patients.

Number	TMA	Patient	Organ	Emerin negative and low intensity (%)	Emerinrich MN/nucleus
16	PCMET2	G-08-WW	lung	55.42635659	0
20	PCMET1	G-12-JM	distant lymph node	0.086580087	0
21	PCMET1	G-12-JM	distant lymph node	0.965250965	0
15	PCMET2	G-08-WW	lung	42.97800338	0.012145749
24	PCMET1	G-15-PZ	penis	56.40394089	0.032258065
5	PCMET2	G-03-NW	lung	17.82841823	0.035897436
6	PCMET2	G-03-NW	lung	35.48387097	0.045454545
13	PCMET2	G-07-SE	bone	2.127659574	0.052631579
22	PCMET1	G-14-MJ	distant lymph node	0	0.057777778
25	PCMET1	G-15-PZ	penis	33.61344538	0.078740157
26	PCMET1	G-15-PZ	urethra	4.141208418	0.114457831
4	PCMET2	G-02-MJ	liver	4.819277108	0.115384615
23	PCMET1	G-14-MJ	distant lymph node	0.354609929	0.189102564
14	PCMET2	G-07-SE	bone	5.637982196	0.19047619
3	PCMET2	G-02-MJ	liver	0.513478819	0.191011236
8	PCMET2	G-04-PJ	distant lymph node	71.48659626	0.192857143
7	PCMET2	G-04-PJ	distant lymph node	74.91448119	0.194630872
12	PCMET2	G-06-RE	lung	18.9296333	0.197802198
11	PCMET2	G-06-RE	lung	12.00564972	0.311594203
27	PCMET1	G-16-SJ	lung	4.395604396	0.381443299
1	PCMET2	G-01-JA	brain	90.25720966	negative
2	PCMET2	G-01-JA	brain	96.50655022	Emerin negative
9	PCMET2	G-05-PS	bone	85.57377049	Emerin negative
10	PCMET2	G-05-PS	bone	92.85714286	Emerin negative
17	PCMET1	G-10-BK	brain	100	Emerin negative
18	PCMET1	G-10-BK	brain	100	Emerin negative
19	PCMET1	G-11-JJ	lung	98.57142857	Emerin negative
28	PCMET1	G-16-SJ	lung	85.14285714	Emerin negative

Supplementary Table 7 Antibody list used in the study.

Target	Clone/Cat.#	Company	Dilution	
			Immunofluorescence staining	Western blotting
**Emerin**	4G5	Novocastra	1:500	1:2000
**Emerin**	PA529731	ThermoFisher Sci.	1:500	1:2000
**Sec61B**	ab244487	abcam	1:100	
**LBR (Lamin B receptor)**	ab232731	abcam	1:200	
**cGAS**	D1D3G	Cell Signaling	1:200	
**SUN2**	ab124916	abcam	1:100	
**LAP2alpha**	3A3	Cell Signaling	1:100	
**BAF-1**	A-11 X	Santa Cruz Biotechnology	1:100	
**γH2AX**	20E3	Cell Signaling	1:200	
**SUN1**	ab124770	abcam	1:100	
**H3K27me3**	C36B11	Cell Signaling	1:100	
**Lamin B1**	ab16048	abcam	1:200	1:2000
**p62**	ab194721	abcam	1:200	
**Lamin A/C**	ab215495	abcam	1:200	
**Lamin A/C**	MA3-1000	ThermoFisher Sci.	1:500	1:2000
**Nesprin 1**	ab192234	abcam	1:100	
**SMC3**	PA5-29131	ThermoFisher Sci.	1:200	1:1000
**BRCA2**	HPA026815	Sigma-Aldrich		1:1000
**XRCC2**	HPA065153	Sigma-Aldrich		1:1000
**Pericentrin**	ab28144	abcam	1:200	
**Pericentrin**	ab270119	abcam	1:200	
**Paxilin**	ab28144	abcam	1:200	
**α-Tubulin**	T5168	Sigma-Aldrich		1:5000
**β-actin**	AC-74	Sigma-Aldrich		1:10000
**Secondary antibodies**				
**Goat anti-Rabbit IgG (H+L) Alexa Fluor™ 488**	A11008	ThermoFisher Sci.	1:500	
**Goat anti-Rabbit IgG (H+L) Alexa Fluor™ 546**	A11010	ThermoFisher Sci.	1:500	
**Goat anti-Rabbit IgG (H+L) Alexa Fluor™ 647**	A21244	ThermoFisher Sci.	1:500	
**Goat anti-Mouse IgG (H+L) Alexa Fluor™ 488**	A11001	ThermoFisher Sci.	1:500	
**Goat anti-Mouse IgG (H+L) Alexa Fluor™ 546**	A11003	ThermoFisher Sci.	1:500	

**Goat anti-Mouse IgG (H+L) Alexa Fluor™ 647**	A21235	ThermoFisher Sci.	1:500	
**AlexaFluor® 680-conjugated AffiniPure Goat Anti-Rabbit**	111-625-144	Jackson ImmunoResearch	-	1:12500
**AlexaFluor® 790-conjugated AffiniPure Donkey Anti-Mouse**	715-655-150	Jackson ImmunoResearch	-	1:12500

Supplementary Table 8 RNAi sequences used in the study

Target Gene Symbol	siRNA ID	Sense	Antisense
**SMC3**	s17427	GCCUAAGCAACGUAGCUUAtt	UAAGCUACGUUGCUUAGCat
**LMNA**	144426	GGAGCUGAAAGCGCGCAAUtt	AUUGCGCGCUUUCAGCUCCtt
**LMNB1**	144054	GCUCUUGCUACUGCACUUGtt	CAAGUGCAGUAGCAAGAGCtg
**BRCA2**	s2085	GGAUUAUACAUAUUUCGCAtt	UGCGAAAUAUGUAUAAUCCag
**XRCC2**	s14945	GGCUAGUUACAAUUCUUGAtt	uCAAGAAUUGUAACUAGCCgg

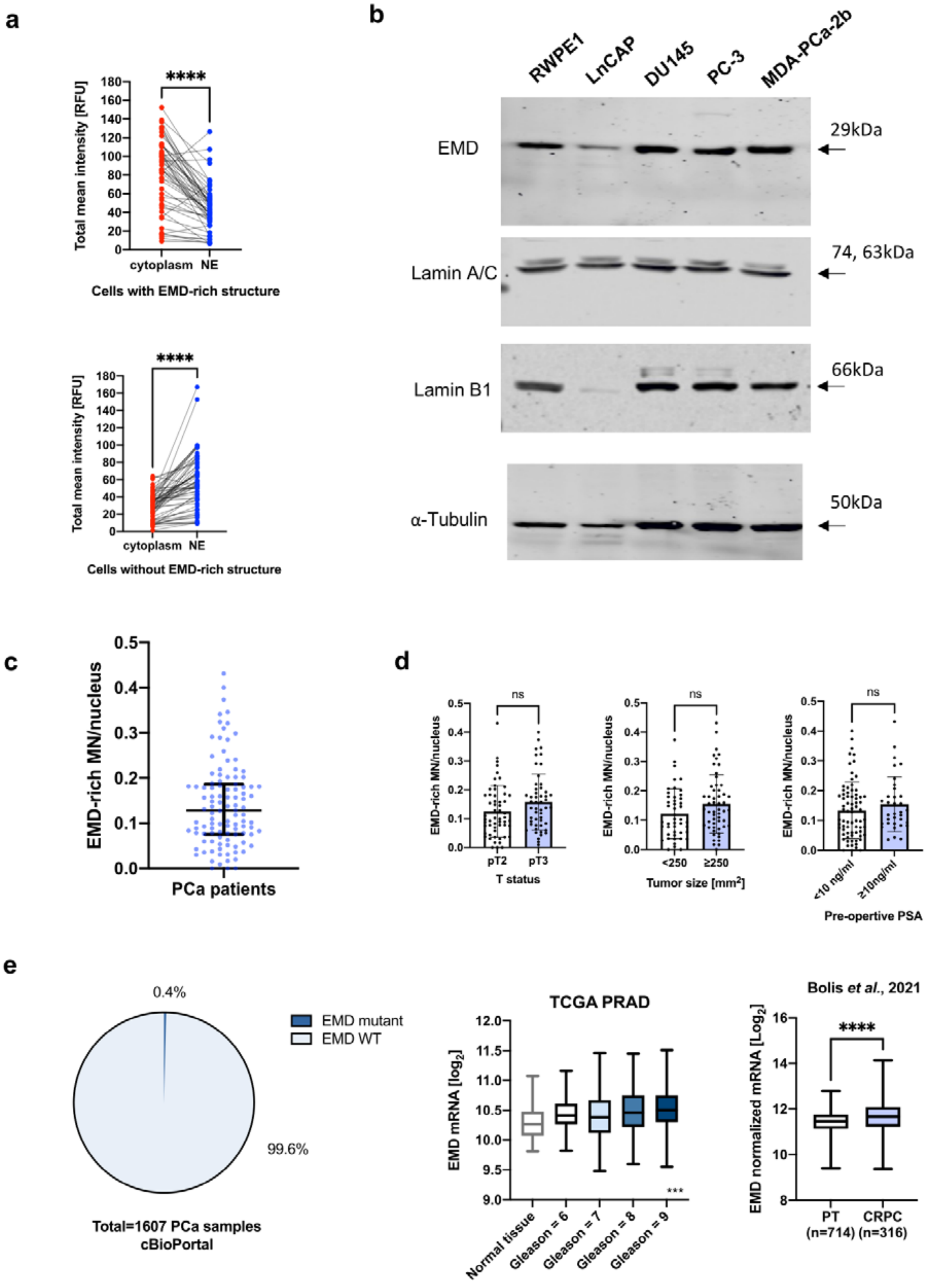
a
****
Total mean intensity [RFU]
cytoplasm NE
Cells with EMD-rich structure
****
Total mean intensity [RFU]
cytoplasm NE
Cells without EMD-rich structure
b
RWPE1
LnCAP
DU145
PC-3
MDA-PCa-2b
EMD
29kDa
Lamin A/C
74, 63kDa
Lamin B1
66kDa
α-Tubulin
50kDa
c
EMD-rich MN/nucleus
PCa patients
d
EMD-rich MN/nucleus
ns
pT2 pT3
T status
ns
<250 ≥250
Tumor size [mm²]
ns
<10 ng/ml ≥10ng/ml
Pre-opertive PSA
e
0.4%
EMD mutant
EMD WT
99.6%
Total=1607 PCa samples
cBioPortal
TCGA PRAD
EMD mRNA [log2]
Normal tissue
Gleason = 6
Gleason = 7
Gleason = 8
Gleason = 9
***
Bolis et al., 2021
EMD normalized mRNA [Log2]
****
PT
(n=714)
CRPC
(n=316)

Supplementary Figure 1

a Total mean Emerin intensity paired measurement in NE comparing to cytoplasm cells with Emerin-rich MN (EMD-rich MN, n=51 cells), and cells without Emerin-rich MN (n-63 cells).

b Western blot performed in various prostate cell lines for Emerin (EMD), Lamin A/C and Lamin B1.

c Emerin-rich structures distribution among PCa patients (n=107).

d Clinical associations between Emerin-rich MN and T status (n=107 patients) tumor size (n=100 patients), and pre-operative PSA levels (n=119 patients).

e Prevalence of EMD mutations in prostate adenocarcinoma according to cBioPortal for Cancer Genomics; association of EMD mRNA expression to Gleason score according to The Cancer Genome Atlas; comparison between EMD mRNA expression in primary prostate cancer tumors (PT) and castration-resistant prostate cancer (CRPC) according to Bolis *et al.*, 2021.

Paired plot: Wilcoxon matched-pair signed rank test; Box-plot; Whiskers indicate min to max values, within the box the first quartile, median, third quartile are represented. For multiple comparison Kruskal-Wallis test was used. Scatter-plot with bar (mean with SD) and bar-plots Mann-Whitney U test. Error bars indicate SD.*P < 0.05; **P < 0.01; ***P < 0.001; ****P < 0.0001; ns, not significant.

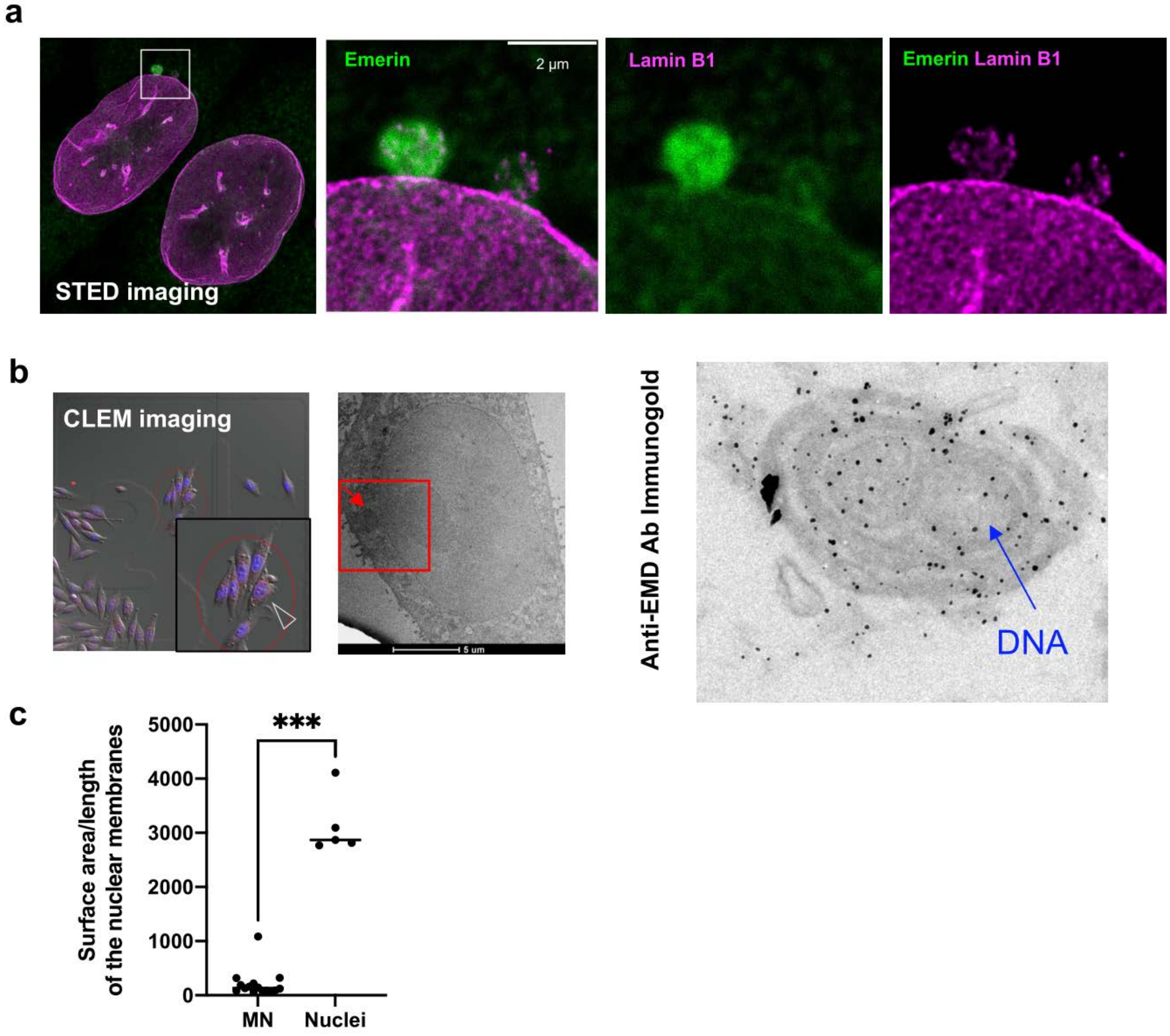

Supplementary Figure 2

a Super Resolution Stimulated Emission Depletion (STED) microscopy of Emerin-rich MN stained for Emerin and Lamin B1, EMD is shown in green, Lamin B1 in magenta.

b Brightfield image and electron microscopy image of a cell in Correlative light electron microscopy (CLEM) experiment of Emerin-rich MN. Left panel, immunogold labeling of Emerin-rich MN.

c The average ratio of surface area to nuclear membranes in micronuclei comparing to the nuclei of neighboring cells that did not have micronuclei.

Scatter-plot with bar (mean with SD) and bar-plots Mann-Whitney U test. Error bars indicate SD.*P < 0.05; **P < 0.01; ***P < 0.001; ****P < 0.0001; ns, not significant.

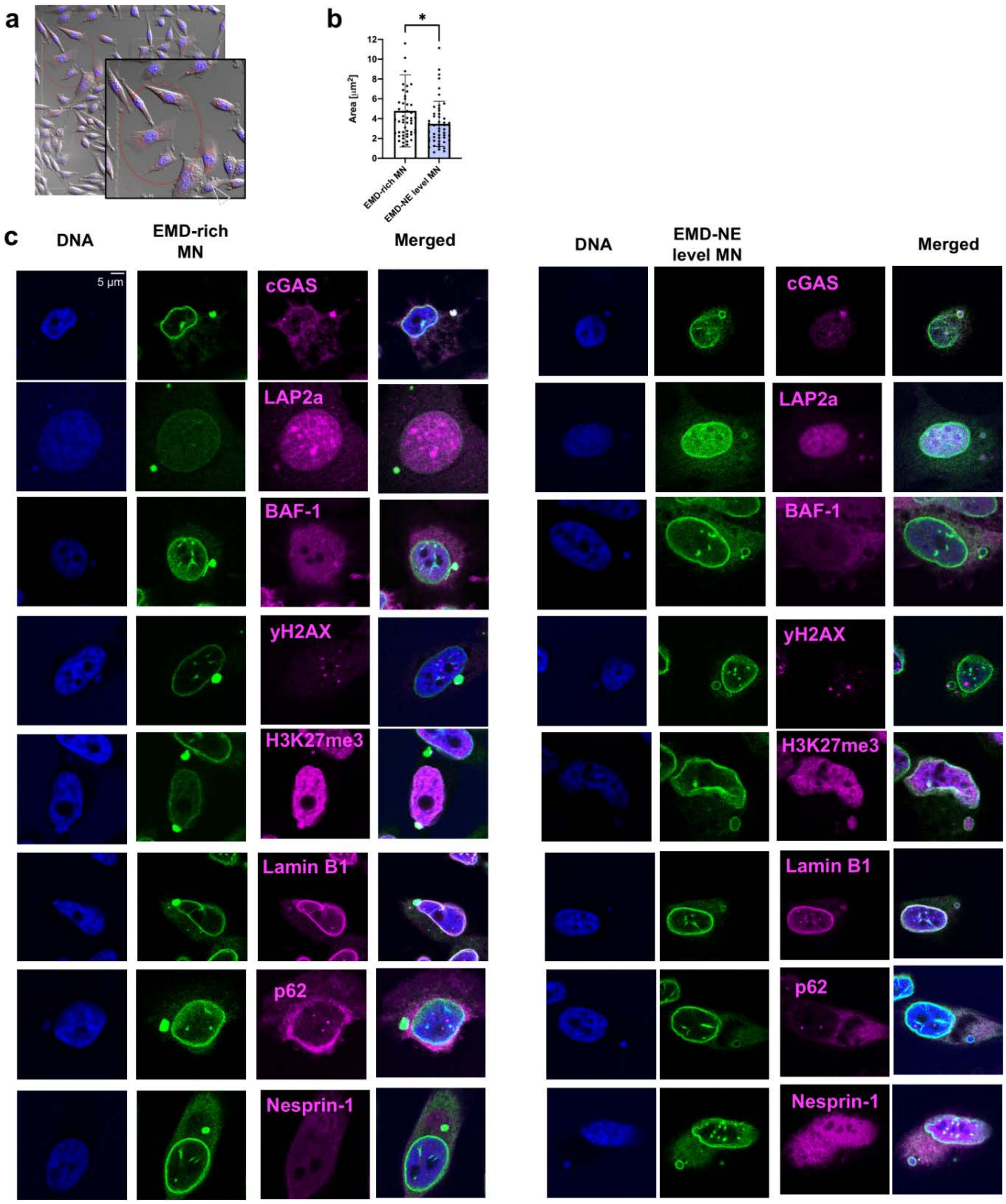

Supplementary Figure 3

a Brightfield image of a cell with Emerin-rich (EMD-rich) MN and Emerin-NE-level MN (EMD-NE-level) that was further used in correlative light electron microscopy (CLEM).

b Area quantification of Emerin-rich MN (n=51 cells) and EMD-NE-level MN (n=50 cells).

c Representative micrographs of staining for cGAS, LAP2a, BAF-1, yX2AX, H3K27me2, Lamin B1, p62, and Nesprin-1 in Emerin-rich MN and Emerin-NE-level MN, EMD is presented in green, other proteins in magenta.

Scatter-plot with bar (mean with SD) and bar-plots Mann-Whitney U test. Error bars indicate SD.*P < 0.05; **P < 0.01; ***P < 0.001; ****P < 0.0001; ns, not significant.

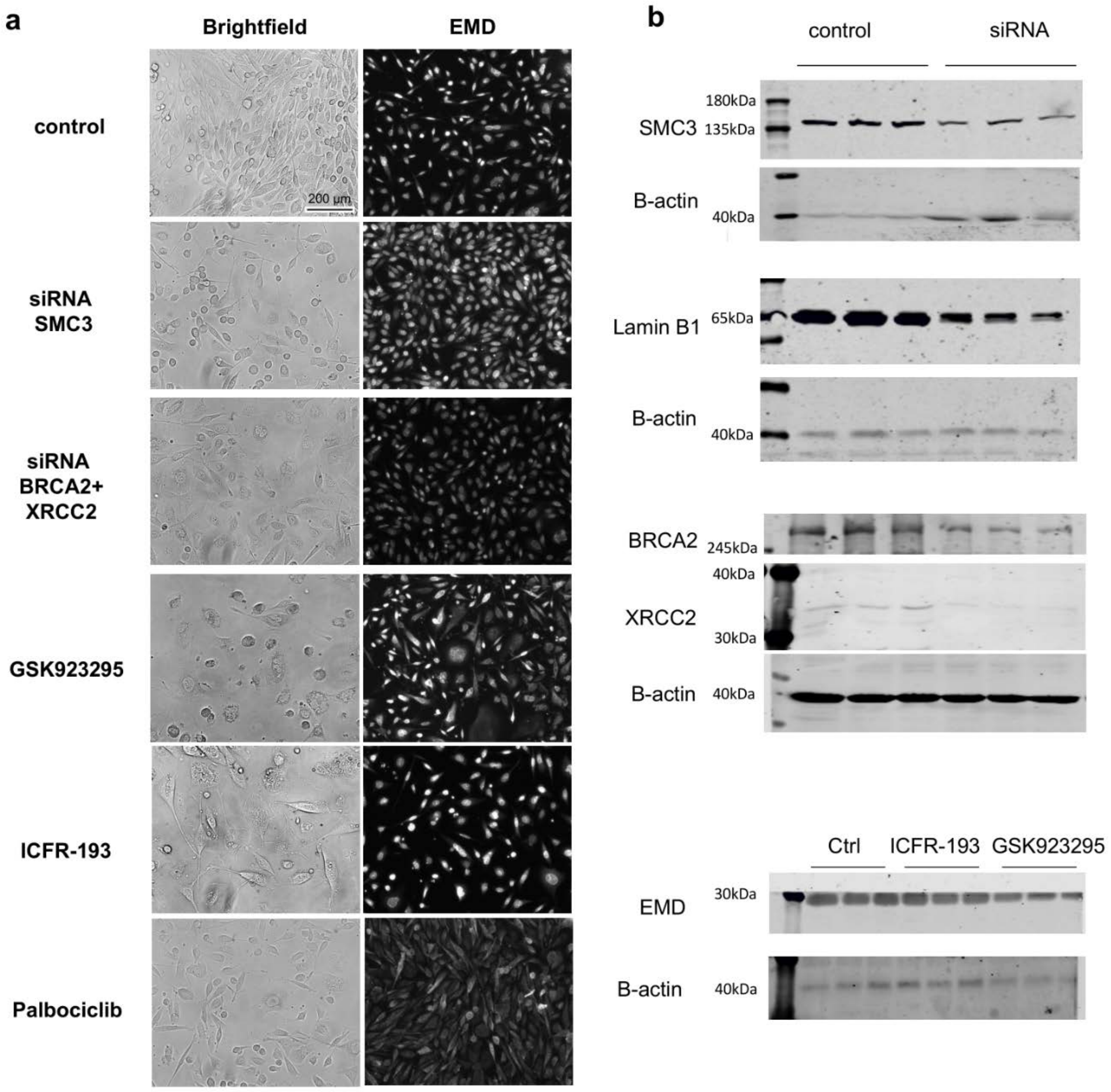

Supplementary Figure 4

a Representative images of brightfield and Emerin in control PC-3 cells and the treated ones.

b Western blot analysis of Emerin in PC-3 cells under various knock-down conditions.

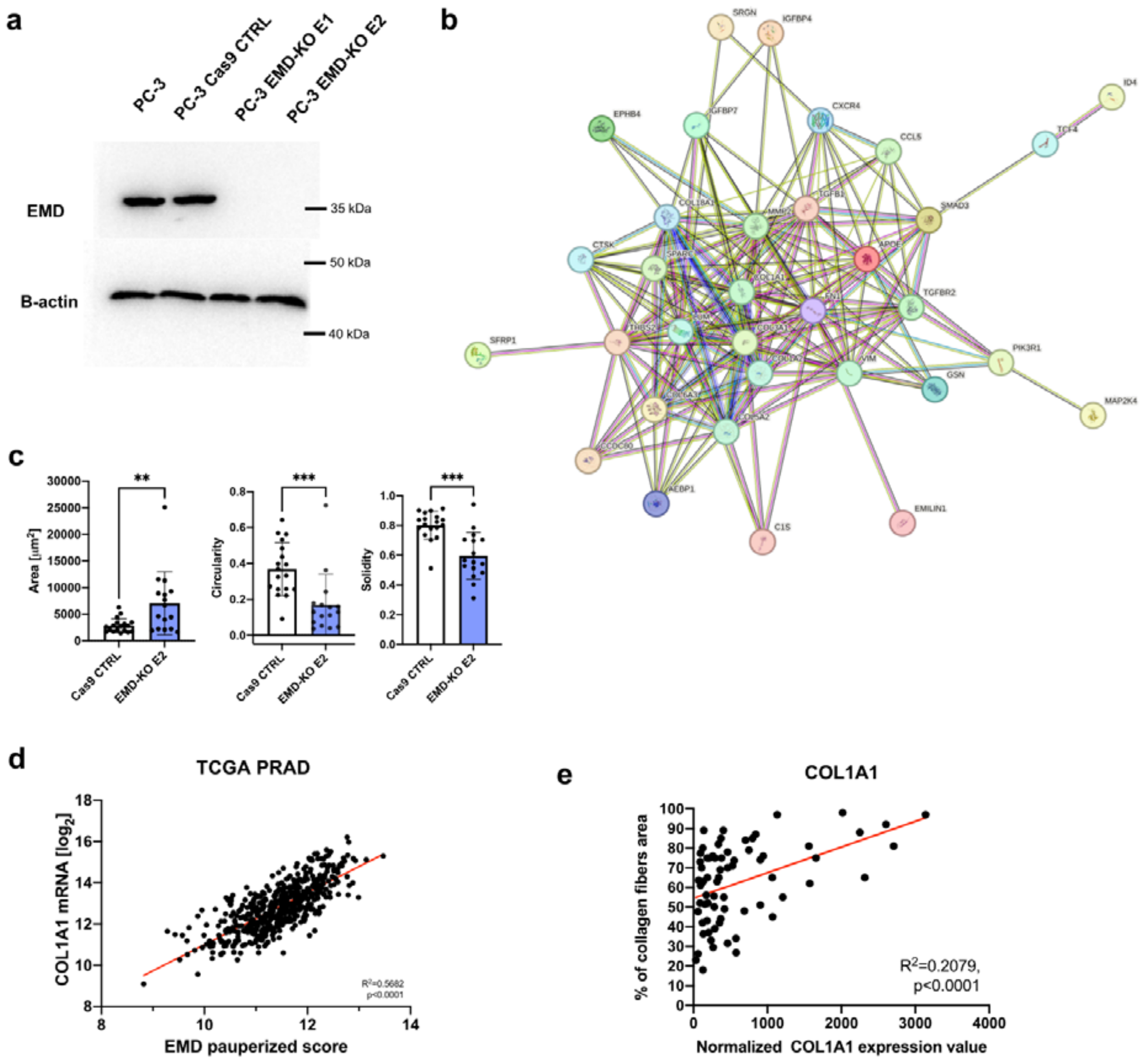

Supplementary Figure 5

a The interaction network of protein products of differentially expressed genes in PCa tumors with Emerin pauperized phenotype visualized using STRING v11.

b Western blot analysis of Emerin in PC-3 control and EMD-KO cells.

c Comparison of properties including area, circularity, solidity of PC-3 control (n=18) and EMD-KO (n=16) spheroids.

d Correlation between COL1A1 mRNA and Emerin pauperized score in TCGA PRAD dataset.

e Correlation between % of collagen fiber area assessed with Picro Sirius stain and normalized COL1A1 expression value.

Scatter-plot with bar (mean with SD), bar-plots Mann-Whitney U test, correlation simple linear regression with 95% confidence intervals. Error bars indicate SD.*P < 0.05; **P < 0.01; ***P < 0.001; ****P < 0.0001; ns, not significant.

Supplementary Movie 1

Live cell imaging of mitosis of PC-3 cells stably expressing EMD-EGFP construct, treated with IRCF-193 inhibitor. The cell division is associated with chromatin bridge formation and EMD-rich MN formation after its resolution. Scale bar 10 µm, time in minutes. EMD-GFP is color coded.

Supplementary Movie 2

Live cell imaging of mitosis of PC-3 cells stably expressing EMD-EGFP construct, treated with IRCF-193 inhibitor. The cell division is associated with chromatin bridge formation and EMD-rich MN formation during its resolution. Scale bar 10 µm, time in minutes. EMD-GFP is color coded.

Supplementary Movie 3

Live cell imaging of PC-3 cells stably expressing EMD-EGFP construct, treated with IRCF-193 inhibitor. The cell division ends up in bi-nucleated cell formation. Scale bar 10 µm, time in minutes. EMD-GFP is color coded.

Supplementary Movie 4

iFRAP (inverse florescence recovery after photobleaching) of nuclear envelope in of PC-3 cells stably expressing EMD-EGFP construct. Scale bar 10 µm, time in seconds.

Supplementary Movie 5

iFRAP (inverse florescence recovery after photobleaching) of EMD-rich MN in of PC-3 cells stably expressing EMD-EGFP construct. Scale bar 10 µm, time in seconds.

Supplementary Movie 6

Comparison of 2D migration properties of PC-3 cells stably expressing EMD-EGFP construct in cell with nuclear envelope localization of EMD and a cell with EMD-rich MN. Scale bar 10 µm, time in minutes.
